# Supplementary material for: Boosting targeted genome editing using the hei-tag
Source: eLife. 2022 Mar 25;11:e70558. doi: 10.7554/eLife.70558 (PMC9068219; doi:10.7554/eLife.70558)
Supplement: Supplementary file 1. [file elife-70558-supp1.docx]

**Figure 1-supplemental file 1**

| Nucleotide and translated amino acid sequence of heiCas9.  **myc**-flexible linker-**oNLS**-Cas9-**oNLS** |
| --- |
| 1 atggagcagaagctgatcagcgaggaggacctgggaggaagcggaccacctcccaagagg 60 |
| 1 M **E Q K L I S E E D L** G G S G **P P P K R** 20 |
| 61 cccaggctggacctcgaggataaaaagtattctattggtttagacatcggcaccaacagc 120 |
| 21 **P R L D** L E D K K Y S I G L D I G T N S 40 |
| 121 gtgggctgggccgtgatcaccgacgagtacaaggtgcccagcaagaaattcaaggtgctg 180 |
| 41 V G W A V I T D E Y K V P S K K F K V L 60 |
| 181 ggcaacaccgacagacacagcatcaagaaaaacctgatcggcgccctgctcttcgactcc 240 |
| 61 G N T D R H S I K K N L I G A L L F D S 80 |
| 241 ggcgaaaccgccgaggccaccagactgaagagaaccgccagacggagatacaccagacgg 300 |
| 81 G E T A E A T R L K R T A R R R Y T R R 100 |
| 301 aagaatagaatctgctacctgcaggagatcttcagcaacgagatggccaaggtggacgat 360 |
| 101 K N R I C Y L Q E I F S N E M A K V D D 120 |
| 361 agcttctttcacagactggaagagagcttcctggtggaagaggacaagaaacacgagaga 420 |
| 121 S F F H R L E E S F L V E E D K K H E R 140 |
| 421 caccccatcttcggcaacatcgtggacgaggtggcctaccacgagaagtaccccaccatc 480 |
| 141 H P I F G N I V D E V A Y H E K Y P T I 160 |
| 481 taccacctgagaaagaaactggtggacagcaccgacaaggccgacctgagactgatctac 540 |
| 161 Y H L R K K L V D S T D K A D L R L I Y 180 |
| 541 ctggcactggcccacatgatcaagttcagaggccacttcctgatcgagggcgacctgaac 600 |
| 181 L A L A H M I K F R G H F L I E G D L N 200 |
| 601 cccgacaacagcgacgtggacaagctgttcatccagctggtgcagacctacaaccagctg 660 |
| 201 P D N S D V D K L F I Q L V Q T Y N Q L 220 |
| 661 ttcgaagagaaccctatcaacgccagcggcgtggacgccaaggccatcctgagcgccaga 720 |
| 221 F E E N P I N A S G V D A K A I L S A R 240 |
| 721 ctcagcaagagcagacggctggagaacctgatcgcccagctgcccggcgagaagaaaaac 780 |
| 241 L S K S R R L E N L I A Q L P G E K K N 260 |
| 781 ggcctgttcggcaacctgatcgccctgagcctgggcctgacccccaacttcaagagcaac 840 |
| 261 G L F G N L I A L S L G L T P N F K S N 280 |
| 841 ttcgacctggccgaggacgccaagctgcagctgagcaaggacacctacgacgatgacctg 900 |
| 281 F D L A E D A K L Q L S K D T Y D D D L 300 |
| 901 gacaacctcctggcccagatcggcgaccagtacgccgacctgttcctcgcagccaagaac 960 |
| 301 D N L L A Q I G D Q Y A D L F L A A K N 320 |
| 961 ctgagcgacgccatcctcctgagcgacatcctcagagtgaacaccgagatcaccaaggct 1020 |
| 321 L S D A I L L S D I L R V N T E I T K A 340 |
| 1021 cccctgagcgccagcatgatcaagagatacgacgagcaccatcaggacctgaccctcctg 1080 |
| 341 P L S A S M I K R Y D E H H Q D L T L L 360 |
| 1081 aaggccctcgtgagacaacagctgcccgagaagtacaaggagatcttctttgaccagagc 1140 |
| 361 K A L V R Q Q L P E K Y K E I F F D Q S 380 |
| 1141 aagaacggctacgccggctacatcgacggaggcgccagtcaggaagagttctacaagttc 1200 |
| 381 K N G Y A G Y I D G G A S Q E E F Y K F 400 |
| 1201 atcaagcccatcctggagaagatggacggcaccgaagagctgctcgtgaagctgaacaga 1260 |
| 401 I K P I L E K M D G T E E L L V K L N R 420 |
| 1261 gaggacctgctcagaaagcagagaaccttcgacaacggcagcatcccccaccagatccac 1320 |
| 421 E D L L R K Q R T F D N G S I P H Q I H 440 |
| 1321 ctgggcgagctgcacgccatcctgagacggcaggaggacttctaccccttcctgaaggac 1380 |
| 441 L G E L H A I L R R Q E D F Y P F L K D 460 |
| 1381 aacagagagaagattgaaaagatcctgaccttcagaatcccctactatgtgggccccctg 1440 |
| 461 N R E K I E K I L T F R I P Y Y V G P L 480 |
| 1441 gccagaggcaacagcagattcgcctggatgaccaggaagagcgaagagacaatcacacct 1500 |
| 481 A R G N S R F A W M T R K S E E T I T P 500 |
| 1501 tggaacttcgaagaggtggtcgacaaaggcgccagcgcccagagcttcatcgagagaatg 1560 |
| 501 W N F E E V V D K G A S A Q S F I E R M 520 |
| 1561 accaacttcgacaagaacctgcccaacgagaaggtgctgcccaagcacagcctcctgtac 1620 |
| 521 T N F D K N L P N E K V L P K H S L L Y 540 |
| 1621 gagtacttcaccgtgtacaacgagctgaccaaggtgaagtacgtgaccgagggcatgaga 1680 |
| 541 E Y F T V Y N E L T K V K Y V T E G M R 560 |
| 1681 aagcctgcctttctgagtggcgagcagaagaaagccatcgtggacctgctcttcaagacc 1740 |
| 561 K P A F L S G E Q K K A I V D L L F K T 580 |
| 1741 aacagaaaagtgaccgtgaagcagctgaaggaggactacttcaagaaaatcgagtgcttc 1800 |
| 581 N R K V T V K Q L K E D Y F K K I E C F 600 |
| 1801 gacagcgtggagatcagcggcgtggaggacagattcaacgccagcctgggcacctaccac 1860 |
| 601 D S V E I S G V E D R F N A S L G T Y H 620 |
| 1861 gacctgctcaagattatcaaagacaaggacttcctggacaacgaagagaacgaggacatc 1920 |
| 621 D L L K I I K D K D F L D N E E N E D I 640 |
| 1921 ctggaggacatcgtgctgacactgaccctcttcgaggacagagagatgatcgaagagaga 1980 |
| 641 L E D I V L T L T L F E D R E M I E E R 660 |
| 1981 ctgaagacctacgcccacctgttcgatgacaaggtgatgaagcagctgaagagacggaga 2040 |
| 661 L K T Y A H L F D D K V M K Q L K R R R 680 |
| 2041 tacaccggctggggcagactgagcagaaagctgatcaacggcatcagagacaagcagagc 2100 |
| 681 Y T G W G R L S R K L I N G I R D K Q S 700 |
| 2101 ggcaagaccatcctggacttcctgaagagcgacggcttcgccaacagaaacttcatgcag 2160 |
| 701 G K T I L D F L K S D G F A N R N F M Q 720 |
| 2161 ctgatccacgatgacagcctgaccttcaaggaggacatccagaaagcccaagtgagcggg 2220 |
| 721 L I H D D S L T F K E D I Q K A Q V S G 740 |
| 2221 cagggcgacagcctgcacgagcatatcgccaacctggctggcagccccgccatcaagaaa 2280 |
| 741 Q G D S L H E H I A N L A G S P A I K K 760 |
| 2281 ggcatcctgcagaccgtgaaggtcgtggacgagctggtcaaggtgatgggcagacacaag 2340 |
| 761 G I L Q T V K V V D E L V K V M G R H K 780 |
| 2341 cccgagaacatcgtgattgagatggccagagagaaccagacaacccagaagggccagaag 2400 |
| 781 P E N I V I E M A R E N Q T T Q K G Q K 800 |
| 2401 aacagcagagagagaatgaagagaatcgaagagggcatcaaggagctgggcagccagatc 2460 |
| 801 N S R E R M K R I E E G I K E L G S Q I 820 |
| 2461 ctgaaggagcaccccgtggagaacacccagctgcagaacgagaagctgtacctgtattac 2520 |
| 821 L K E H P V E N T Q L Q N E K L Y L Y Y 840 |
| 2521 ctgcagaacggcagagacatgtacgtggaccaggagctggacatcaacagactgagcgat 2580 |
| 841 L Q N G R D M Y V D Q E L D I N R L S D 860 |
| 2581 tacgacgtggatcacatcgtcccccagagcttcctgaaggatgacagcatcgataacaag 2640 |
| 861 Y D V D H I V P Q S F L K D D S I D N K 880 |
| 2641 gtgctgaccagaagcgacaagaacagaggcaagagcgacaacgtgcccagcgaagaggtc 2700 |
| 881 V L T R S D K N R G K S D N V P S E E V 900 |
| 2701 gtgaagaaaatgaagaactactggagacagctcctgaacgccaagctgatcacccagaga 2760 |
| 901 V K K M K N Y W R Q L L N A K L I T Q R 920 |
| 2761 aagttcgacaacctgaccaaggccgagagaggcgggctgagcgagctcgacaaagccggc 2820 |
| 921 K F D N L T K A E R G G L S E L D K A G 940 |
| 2821 ttcatcaagagacagctggtggaaaccagacagatcaccaagcacgtggcccagatcctg 2880 |
| 941 F I K R Q L V E T R Q I T K H V A Q I L 960 |
| 2881 gacagcagaatgaacaccaagtacgacgagaacgataagctgatcagagaggtgaaggtc 2940 |
| 961 D S R M N T K Y D E N D K L I R E V K V 980 |
| 2941 atcaccctgaagagcaaactggtgagcgacttcagaaaggacttccagttctacaaggtg 3000 |
| 981 I T L K S K L V S D F R K D F Q F Y K V 1000 |
| 3001 agagagatcaataactaccatcacgctcatgacgcctacctgaacgccgtcgtgggcacc 3060 |
| 1001 R E I N N Y H H A H D A Y L N A V V G T 1020 |
| 3061 gccctgatcaagaaataccccaagctggagagcgagttcgtgtacggcgactacaaggtg 3120 |
| 1021 A L I K K Y P K L E S E F V Y G D Y K V 1040 |
| 3121 tacgacgtgagaaagatgatcgccaagagcgagcaggagatcggcaaggccaccgccaag 3180 |
| 1041 Y D V R K M I A K S E Q E I G K A T A K 1060 |
| 3181 tacttcttttacagcaacatcatgaacttctttaagaccgagatcaccctggccaacggc 3240 |
| 1061 Y F F Y S N I M N F F K T E I T L A N G 1080 |
| 3241 gagatcagaaagaggcccctgatcgaaaccaacggcgaaaccggcgagatcgtgtgggac 3300 |
| 1081 E I R K R P L I E T N G E T G E I V W D 1100 |
| 3301 aagggcagagacttcgccaccgtgagaaaggtgctgagcatgccccaggtgaacatcgtg 3360 |
| 1101 K G R D F A T V R K V L S M P Q V N I V 1120 |
| 3361 aagaaaaccgaggtgcagaccggaggcttcagcaaggagagcatcctgcccaagagaaac 3420 |
| 1121 K K T E V Q T G G F S K E S I L P K R N 1140 |
| 3421 agcgacaagctgatcgccagaaagaaagactgggaccccaagaaatacggaggcttcgac 3480 |
| 1141 S D K L I A R K K D W D P K K Y G G F D 1160 |
| 3481 agccccaccgtggcctacagcgtgctggtcgtggccaaggtggagaagggcaagagcaag 3540 |
| 1161 S P T V A Y S V L V V A K V E K G K S K 1180 |
| 3541 aaactgaagagcgtgaaggagctcctgggcatcaccatcatggagagatccagcttcgag 3600 |
| 1181 K L K S V K E L L G I T I M E R S S F E 1200 |
| 3601 aagaaccccatcgacttcctggaggccaagggctacaaggaggtgaagaaagacctgatt 3660 |
| 1201 K N P I D F L E A K G Y K E V K K D L I 1220 |
| 3661 atcaagctgcccaagtacagcctgttcgagctggagaacggcagaaagagaatgctggcc 3720 |
| 1221 I K L P K Y S L F E L E N G R K R M L A 1240 |
| 3721 agcgccggcgagctgcagaagggcaacgagctggccctgcccagcaagtacgtgaacttc 3780 |
| 1241 S A G E L Q K G N E L A L P S K Y V N F 1260 |
| 3781 ctgtacctggccagccactacgagaagctgaagggcagccccgaggacaacgagcagaag 3840 |
| 1261 L Y L A S H Y E K L K G S P E D N E Q K 1280 |
| 3841 cagctgttcgtggagcagcacaagcactacctggacgaaatcattgagcagatcagcgag 3900 |
| 1281 Q L F V E Q H K H Y L D E I I E Q I S E 1300 |
| 3901 ttcagtaagagagtgatcctggctgacgccaacctggacaaggtgctgagcgcctacaac 3960 |
| 1301 F S K R V I L A D A N L D K V L S A Y N 1320 |
| 3961 aagcacagagacaagcccatcagagagcaggccgagaacatcattcacctgttcaccctg 4020 |
| 1321 K H R D K P I R E Q A E N I I H L F T L 1340 |
| 4021 accaacctgggcgcacccgcagccttcaagtacttcgacaccacaatcgacagaaagaga 4080 |
| 1341 T N L G A P A A F K Y F D T T I D R K R 1360 |
| 4081 tacaccagcaccaaggaggtgctggacgccaccctgatccaccagagcatcaccggcctg 4140 |
| 1361 Y T S T K E V L D A T L I H Q S I T G L 1380 |
| 4141 tacgaaaccagaatcgacctgtcacagcttgggggtgacggatcccctcctcccaagagg 4200 |
| 1381 Y E T R I D L S Q L G G D G S **P P P K R** 1400 |
| 4201 cccaggctggactaa 4215 |
| 1401 **P R L D** * 1404 |
|  |
