# Supplementary material for: Boosting targeted genome editing using the hei-tag
Source: eLife. 2022 Mar 25;11:e70558. doi: 10.7554/eLife.70558 (PMC9068219; doi:10.7554/eLife.70558)
Supplement: Supplementary file 2. — M, cMyc-tag; O, optimized nuclear localization signal (NLS) (Inoue et al., 2016); S, SV40 NLS (Kalderon et al., 1984); Xl, bipartite Xenopus laevis nucleoplasmin NLS (Dingwall et al., 1988). [file elife-70558-supp2.docx]

**Figure 1-figure supplement 1-supplemental file 1**

Sequences of peptide tags fused to mammalian SpCas9 (cf. Figure 1-supplemental file 1)

MFO-Cas9-O (heiCas9)

**myc**-flexible linker-**oNLS**-Cas9-**oNLS**

| 1 atggagcagaagctgatcagcgaggaggacctgggaggaagcggaccacctcccaagagg 60 |
| --- |
| 1 M **E Q K L I S E E D L** G G S G **P P P K R** 20 |
| 61 cccaggctggac-------Cas9-------cctcctcccaagaggcccaggctggactaa 4215 |
| 21 **P R L D** -------CAS9-------**P P P K R** **P R L D** * 1404 |

MIS-Cas9-S

**c-myc**-internal linker-**SV40 NLS**-Cas9- **SV40NLS**

| 1 atg**gagcagaagctgatcagcgaggaggacctg**ggtatccacggagtcccagcagcc**gct** 60 |
| --- |
| 1 M **E Q K L I S E E D L** G I H G V P A A **A**  20 |
| 61 **ccaaagaagaagcgtaaggta**----Cas9----**ccaaagaagaagcgtaaggta**gattaa 4221 |
| 21 **P K K K R K V** ----CAS9----**P K K K R K V** D * 1406 |

MSF-Cas9-S

**c-myc**-**SV40 NLS**-flexible linker-Cas9-**SV40 NLS**

| 1 atg**gagcagaagctgatcagcgaggaggacctg**atg**gctccaaagaagaagcgtaaggta** 60 |
| --- |
| 1 M **E Q K L I S E E D L** M **A P K K K R K V**  20 |
| 61 ggaggaagcgga-------Cas9-------**gctccaaagaagaagcgtaaggta**gattaa 4212 |
| 21 G G S G -------CAS9-------**A P K K K R** **K V** D * 1403 |

MSI-Cas9-Xl (myc-Cas9)

**c-myc**-**Sv40**-internal linker-Cas9-**Xenopus laevis nucleoplasmin NLS**

| 1 atg**gagcagaagctgatcagcgaggaggacctg**atg**gccccaaagaagaagcggaaggtc** 60 |
| --- |
| 1 M **E Q K L I S E E D L** M **A P K K K R K V** |
| 61 ggtatccacggagtcccagcagcc----Cas9----**aaaaggccggcggccacgaaaaag** 4209 |
| 21 G I H G V P A A ----CAS9----**K R P A A T K K** 1403 |
| 4210 **gccggccaggcaaaaaagaaaaag**taa 4236 |
| 1404 **A G Q A K K K K** * 1411 |

OMF-Cas9-O

| **oNLS-c-myc-flexible linker-Cas9-oNLS** |
| --- |
| 1 atg**ccacctcccaagaggcccaggctggac**ctcgag**gagcagaagctgatcagcgaggag** 60 |
| 1 M **P P P K R P R L D** L E **E Q K L I S E E** 20 |
| 61 **gacctg**ggaggaagcgga----Cas9---**cctcctcccaagaggcccaggctggac**taa 4215 |
| 21 **D L** G G S G ----CAS9---**P P P K R** **P R L D** * 1404 |

SMF-Cas9-S

**SV40 NLS**-**c-myc**-flexible linker-Cas9-**SV40 NLS**

| 1 atg**gctccaaagaagaagcgtaaggta**ctcgag**gagcagaagctgatcagcgaggaggac** 60 |
| --- |
| 1 M **A P K K K R K V** L E **E Q K L I S E E D**  20 |
| 61 **ctg**ggaggaagcgga-----Cas9------**gctccaaagaagaagcgtaaggta**gattaa 4215 |
| 21 **L** G G S G -----CAS9------**A P K K K** **R K V** D * 1404 |
