# Supplementary material for: Boosting targeted genome editing using the hei-tag
Source: eLife. 2022 Mar 25;11:e70558. doi: 10.7554/eLife.70558 (PMC9068219; doi:10.7554/eLife.70558)
Supplement: Supplementary file 3. — Sequences and abundance of all locus-mapped reads per replicate (pool) and locus (OlOca2, rx2, rx3, cryaa) of multiplexing with either zCas9 or heiCas9 mRNA injections (Figure 2). Sequences of allele variants (with more than 100 reads) displayed. [file elife-70558-supp3.pdf]

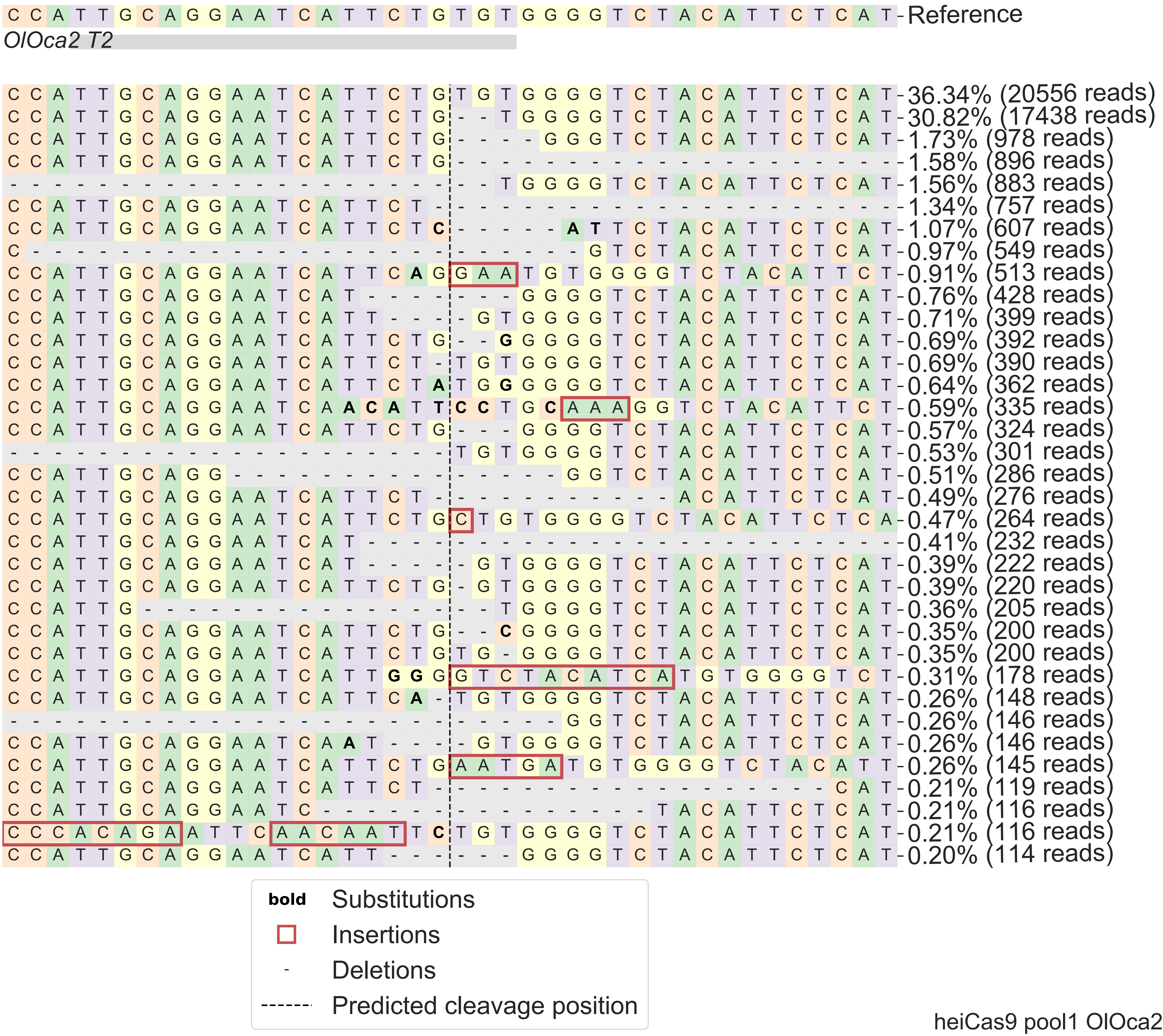

C C A T T G C A G G A A T C A T T C T G T G T G G G G T C T A C A T T C T C A T Reference
OlOca2 T2
36.34% (20556 reads)
30.82% (17438 reads)
1.73% (978 reads)
1.58% (896 reads)
1.56% (883 reads)
1.34% (757 reads)
1.07% (607 reads)
0.97% (549 reads)
0.91% (513 reads)
0.76% (428 reads)
0.71% (399 reads)
0.69% (392 reads)
0.69% (390 reads)
0.64% (362 reads)
0.59% (335 reads)
0.57% (324 reads)
0.53% (301 reads)
0.51% (286 reads)
0.49% (276 reads)
0.47% (264 reads)
0.41% (232 reads)
0.39% (222 reads)
0.39% (220 reads)
0.36% (205 reads)
0.35% (200 reads)
0.35% (200 reads)
0.31% (178 reads)
0.26% (148 reads)
0.26% (146 reads)
0.26% (146 reads)
0.26% (145 reads)
0.21% (119 reads)
0.21% (116 reads)
0.21% (116 reads)
0.20% (114 reads)
bold Substitutions
Insertions
Deletions
Predicted cleavage position
heiCas9 pool1 OlOca2

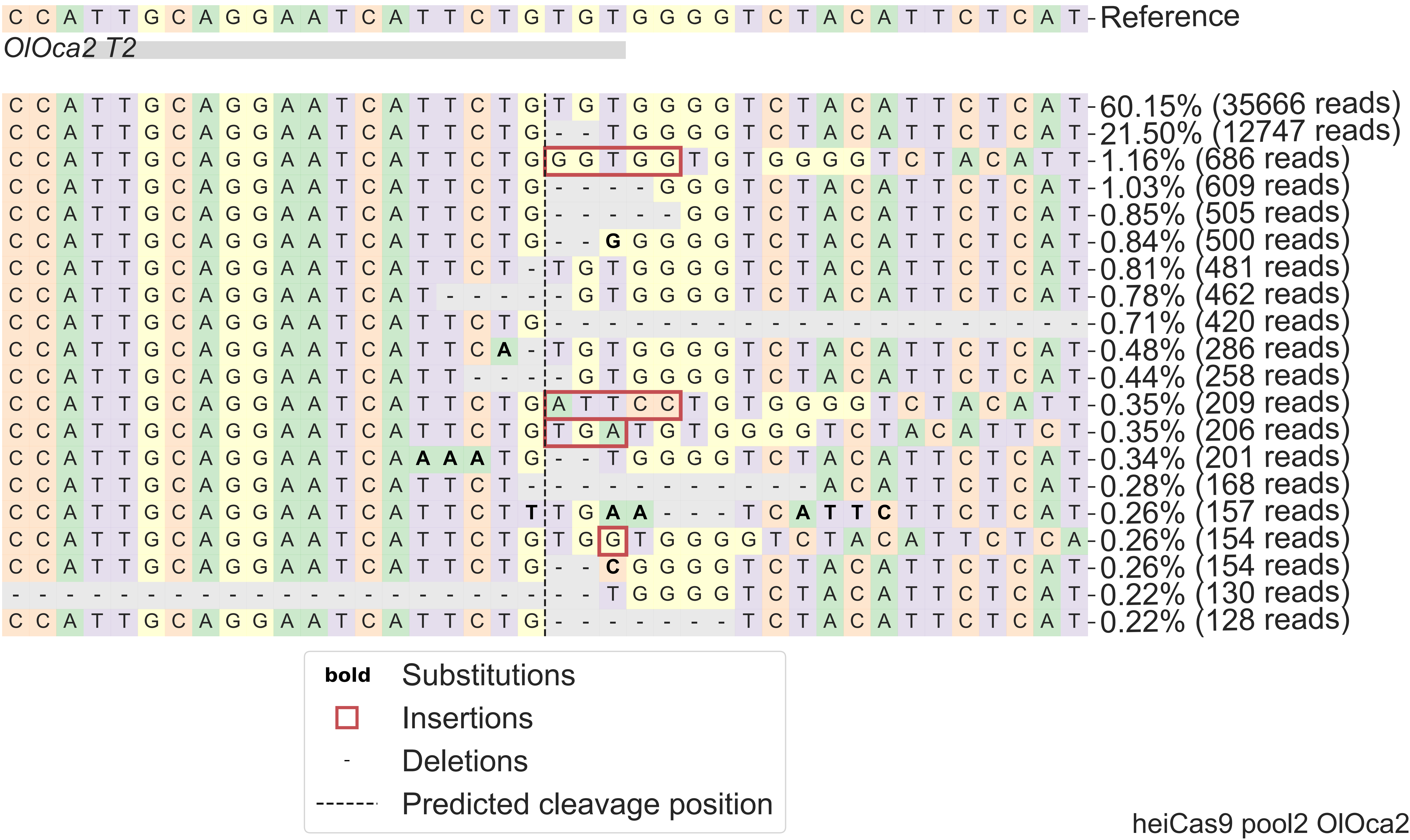

CCATTGCAGGAATCATTCTGTGTGGGGTCTACATTCTCAT Reference
OlOca2 T2
CCATTGCAGGAATCATTCTGTGTGGGGTCTACATTCTCAT 60.15% (35666 reads)
CCATTGCAGGAATCATTCTG--TGGGGTCTACATTCTCAT 21.50% (12747 reads)
CCATTGCAGGAATCATTCTGGGTGGTGTGGGGTCTACATT 1.16% (686 reads)
CCATTGCAGGAATCATTCTG----GGGTCTACATTCTCAT 1.03% (609 reads)
CCATTGCAGGAATCATTCTG-----GGTCTACATTCTCAT 0.85% (505 reads)
CCATTGCAGGAATCATTCTG--GGGGGTCTACATTCTCAT 0.84% (500 reads)
CCATTGCAGGAATCATTCT-TGTGGGGTCTACATTCTCAT 0.81% (481 reads)
CCATTGCAGGAATCAT-----GTGGGGTCTACATTCTCAT 0.78% (462 reads)
CCATTGCAGGAATCATTCTG-------------------- 0.71% (420 reads)
CCATTGCAGGAATCATTCA-TGTGGGGTCTACATTCTCAT 0.48% (286 reads)
CCATTGCAGGAATCATT----GTGGGGTCTACATTCTCAT 0.44% (258 reads)
CCATTGCAGGAATCATTCTGATTCCTGTGGGGTCTACATT 0.35% (209 reads)
CCATTGCAGGAATCATTCTGTGATGTGGGGTCTACATTCT 0.35% (206 reads)
CCATTGCAGGAATCAAAATG--TGGGGTCTACATTCTCAT 0.34% (201 reads)
CCATTGCAGGAATCATTCT-----------ACATTCTCAT 0.28% (168 reads)
CCATTGCAGGAATCATTCTTTGAA---TCATTCTTCTCAT 0.26% (157 reads)
CCATTGCAGGAATCATTCTGTGGTGGGGTCTACATTCTCA 0.26% (154 reads)
CCATTGCAGGAATCATTCTG--CGGGGTCTACATTCTCAT 0.26% (154 reads)
----------------------TGGGGTCTACATTCTCAT 0.22% (130 reads)
CCATTGCAGGAATCATTCTG-------TCTACATTCTCAT 0.22% (128 reads)
bold Substitutions
Insertions
Deletions
Predicted cleavage position
heiCas9 pool2 OlOca2

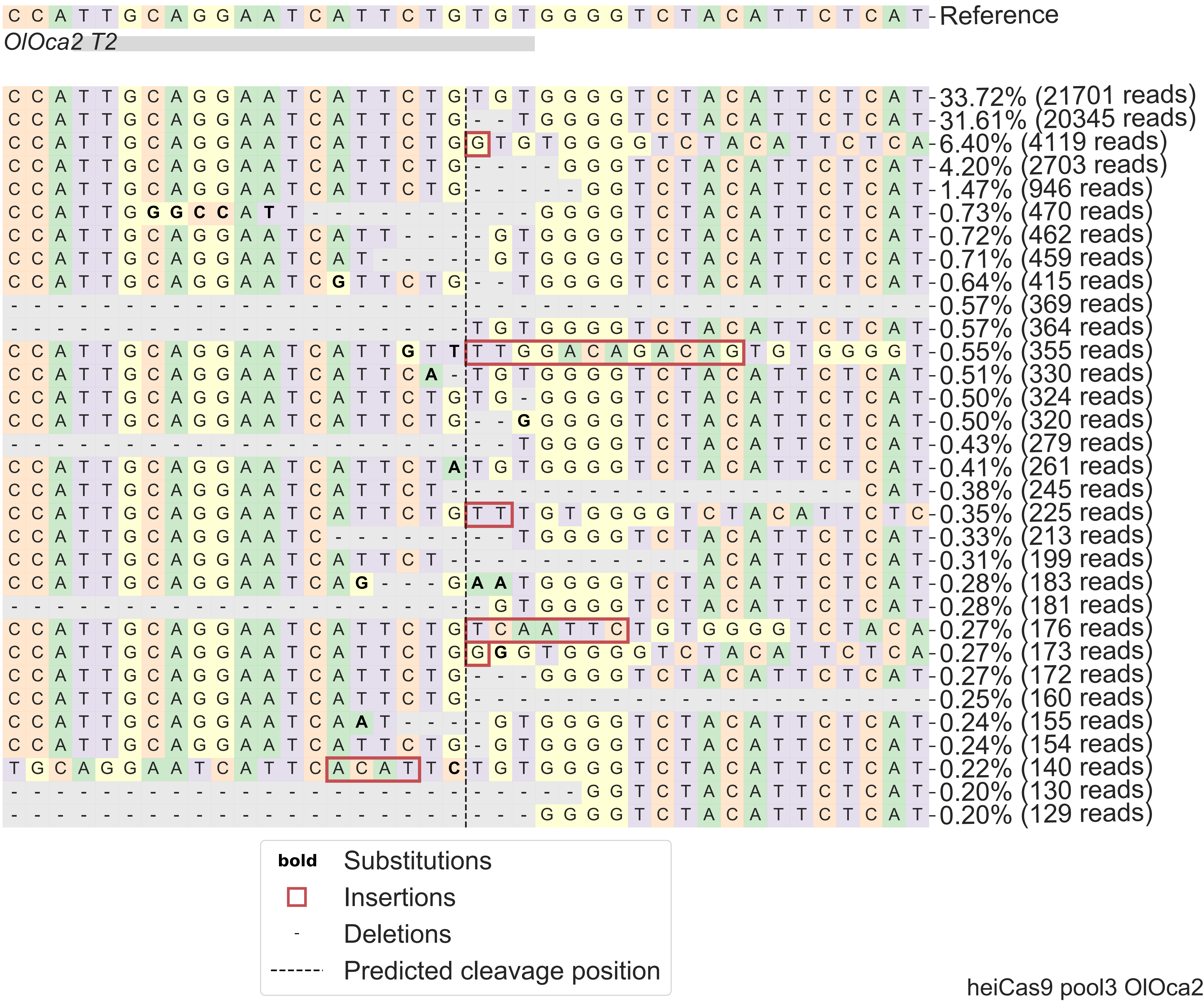

heiCas9 pool3 OlOca2

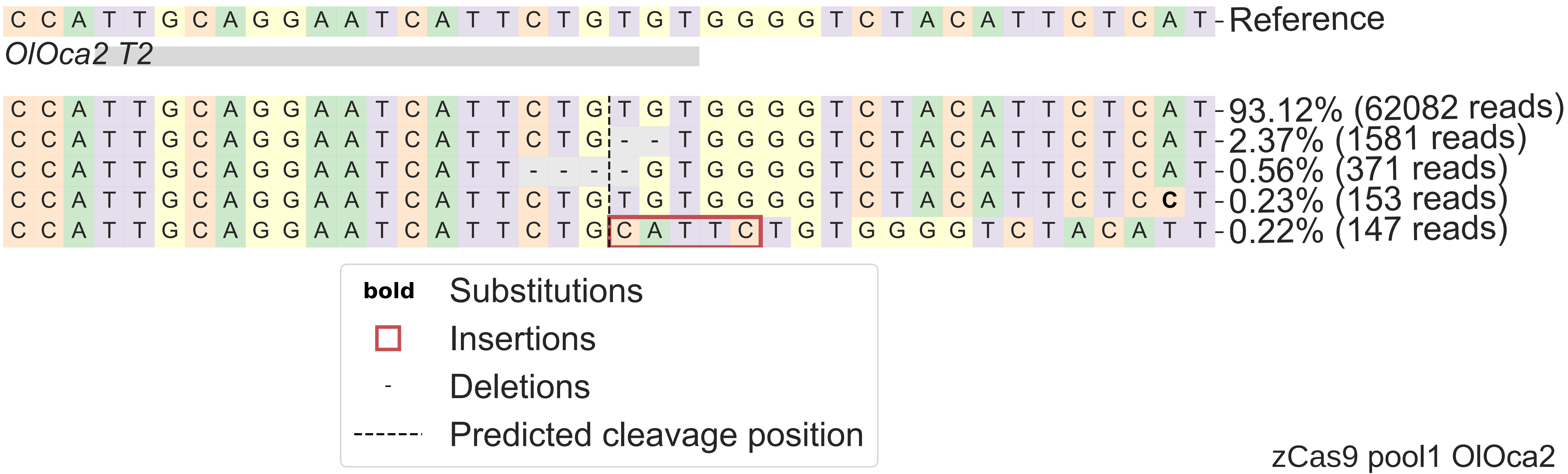
C C A T T G C A G G A A T C A T T C T G T G T G G G G T C T A C A T T C T C A T Reference
OlOca2 T2
C C A T T G C A G G A A T C A T T C T G T G T G G G G T C T A C A T T C T C A T 93.12% (62082 reads)
C C A T T G C A G G A A T C A T T C T G - - T G G G G T C T A C A T T C T C A T 2.37% (1581 reads)
C C A T T G C A G G A A T C A T T - - - - G T G G G G T C T A C A T T C T C A T 0.56% (371 reads)
C C A T T G C A G G A A T C A T T C T G T G T G G G G T C T A C A T T C T C C T 0.23% (153 reads)
C C A T T G C A G G A A T C A T T C T G C A T T C T G T G G G G T C T A C A T T 0.22% (147 reads)
bold Substitutions
Insertions
- Deletions
Predicted cleavage position
zCas9 pool1 OlOca2

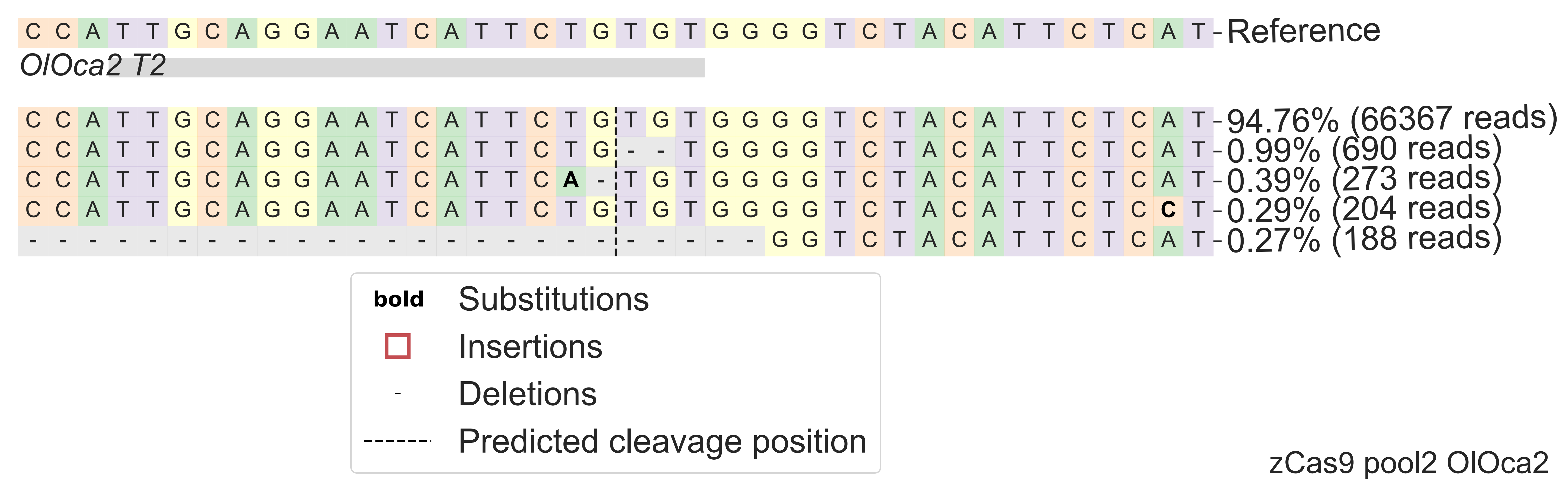
CCATTGCAGGAATCATTCTGTGTGGGGTCTACATTCTCAT Reference
OlOca2 T2
CCATTGCAGGAATCATTCTGTGTGGGGTCTACATTCTCAT 94.76% (66367 reads)
CCATTGCAGGAATCATTCTG--TGGGGTCTACATTCTCAT 0.99% (690 reads)
CCATTGCAGGAATCATTCA-TGTGGGGTCTACATTCTCAT 0.39% (273 reads)
CCATTGCAGGAATCATTCTGTGTGGGGTCTACATTCTCCT 0.29% (204 reads)
-------------------------GGTCTACATTCTCAT 0.27% (188 reads)
bold Substitutions
Insertions
- Deletions
Predicted cleavage position
zCas9 pool2 OlOca2

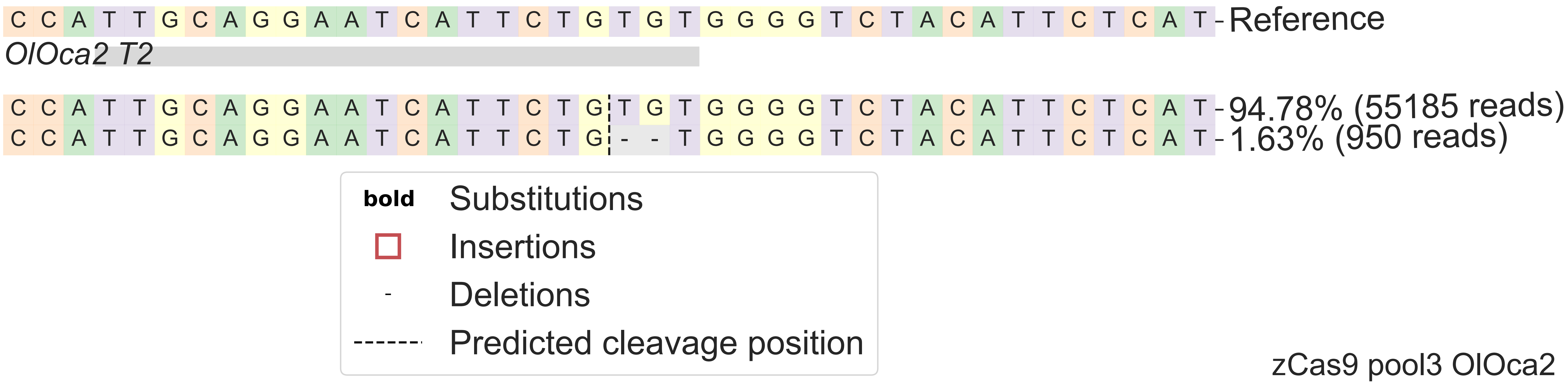

C C A T T G C A G G A A T C A T T C T G T G T G G G G T C T A C A T T C T C A T
Reference
OlOca2 T2
C C A T T G C A G G A A T C A T T C T G T G T G G G G T C T A C A T T C T C A T
94.78% (55185 reads)
C C A T T G C A G G A A T C A T T C T G - - T G G G G T C T A C A T T C T C A T
1.63% (950 reads)
bold Substitutions
Insertions
- Deletions
Predicted cleavage position
zCas9 pool3 OlOca2

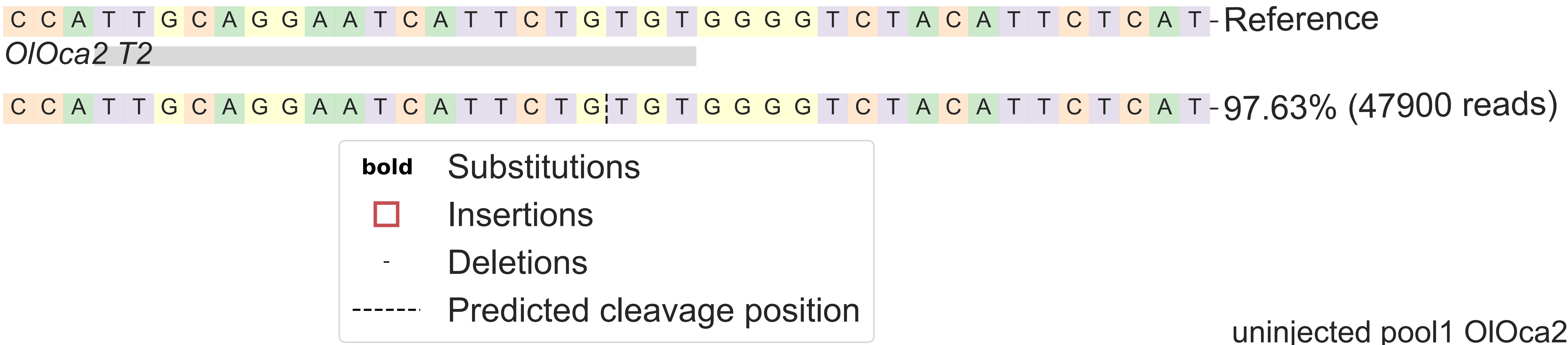
C C A T T G C A G G A A T C A T T C T G T G T G G G G T C T A C A T T C T C A T - Reference
OlOca2 T2
C C A T T G C A G G A A T C A T T C T G T G T G G G G T C T A C A T T C T C A T - 97.63% (47900 reads)
bold Substitutions
Insertions
Deletions
Predicted cleavage position
uninjected pool1 OlOca2

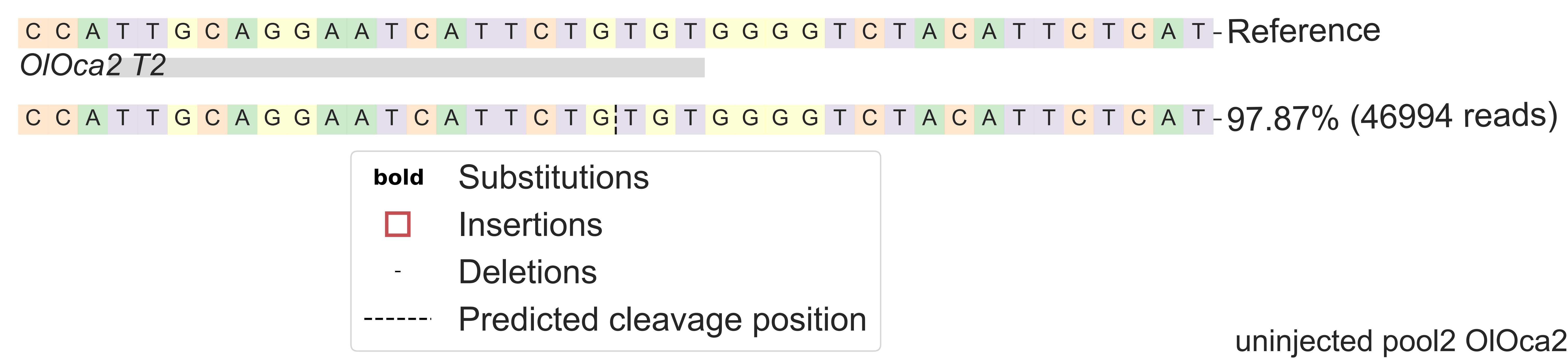
C C A T T G C A G G A A T C A T T C T G T G T G G G G T C T A C A T T C T C A T -
Reference
OlOca2 T2
C C A T T G C A G G A A T C A T T C T G T G T G G G G T C T A C A T T C T C A T -
97.87% (46994 reads)
bold
Substitutions
Insertions
-
Deletions
Predicted cleavage position
uninjected pool2 OlOca2

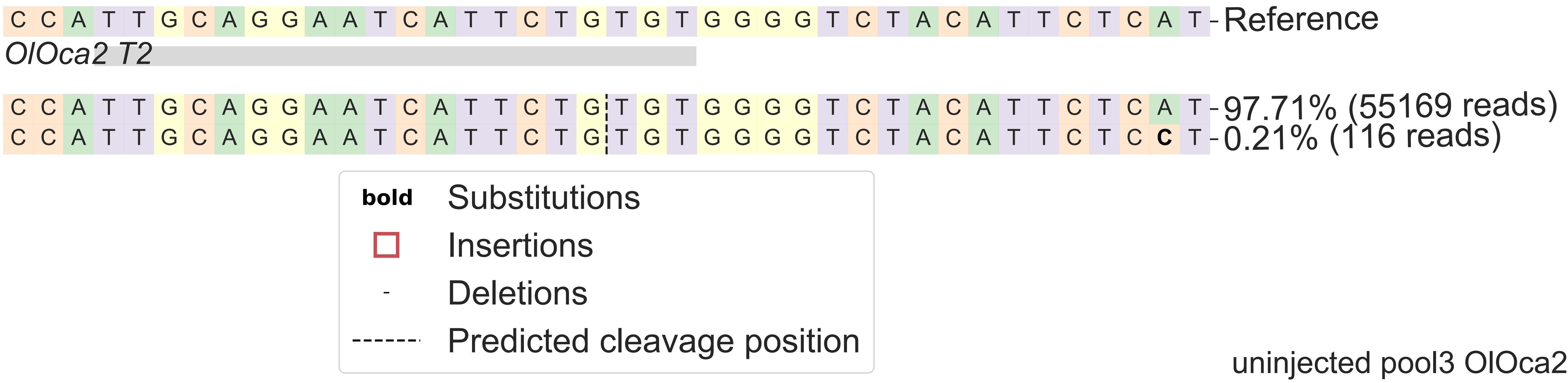
C C A T T G C A G G A A T C A T T C T G T G T G G G G T C T A C A T T C T C A T
Reference
OlOca2 T2
C C A T T G C A G G A A T C A T T C T G T G T G G G G T C T A C A T T C T C A T
97.71% (55169 reads)
C C A T T G C A G G A A T C A T T C T G T G T G G G G T C T A C A T T C T C C T
0.21% (116 reads)
bold Substitutions
Insertions
- Deletions
Predicted cleavage position
uninjected pool3 OlOca2

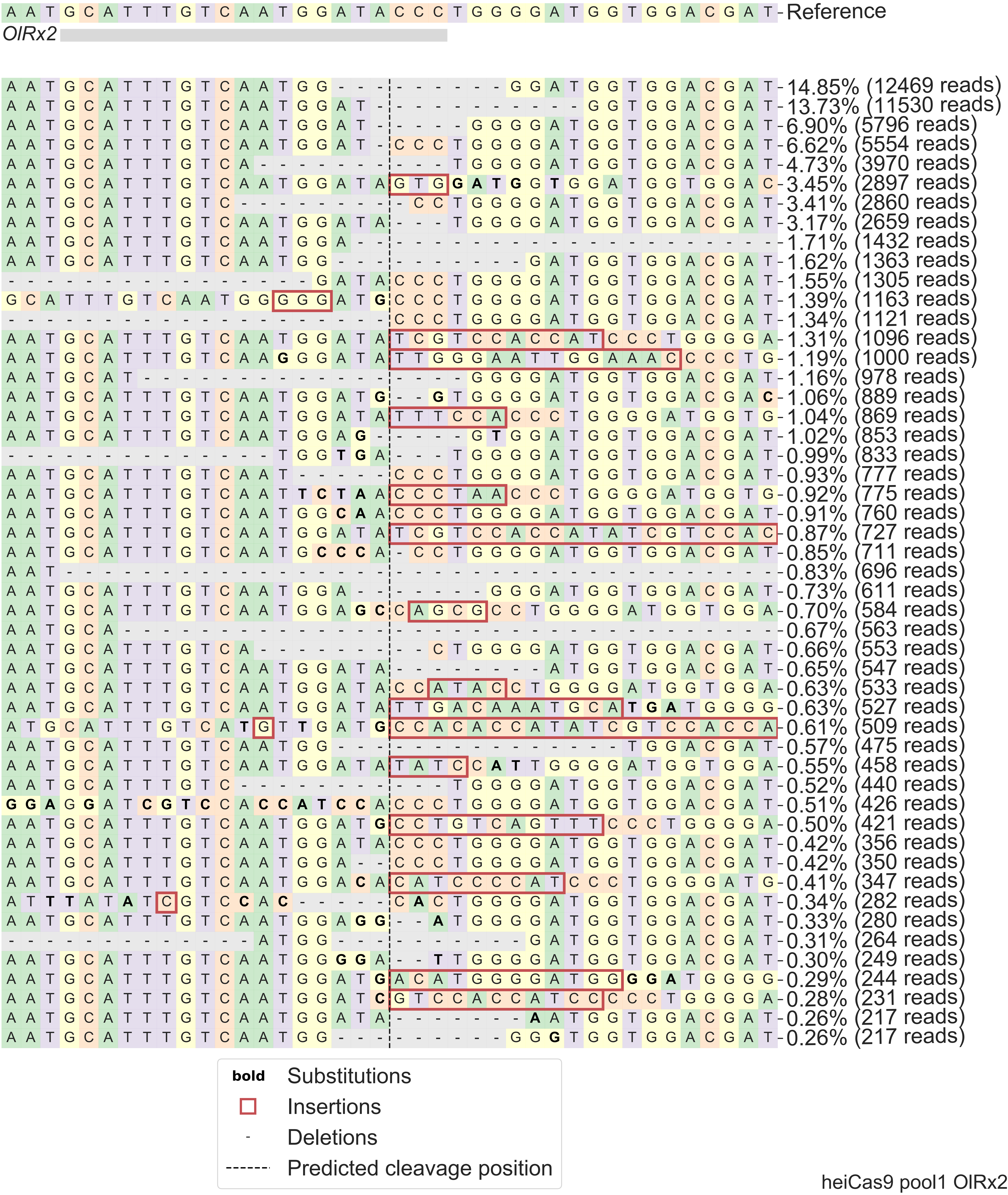

heiCas9 pool1 OlRx2

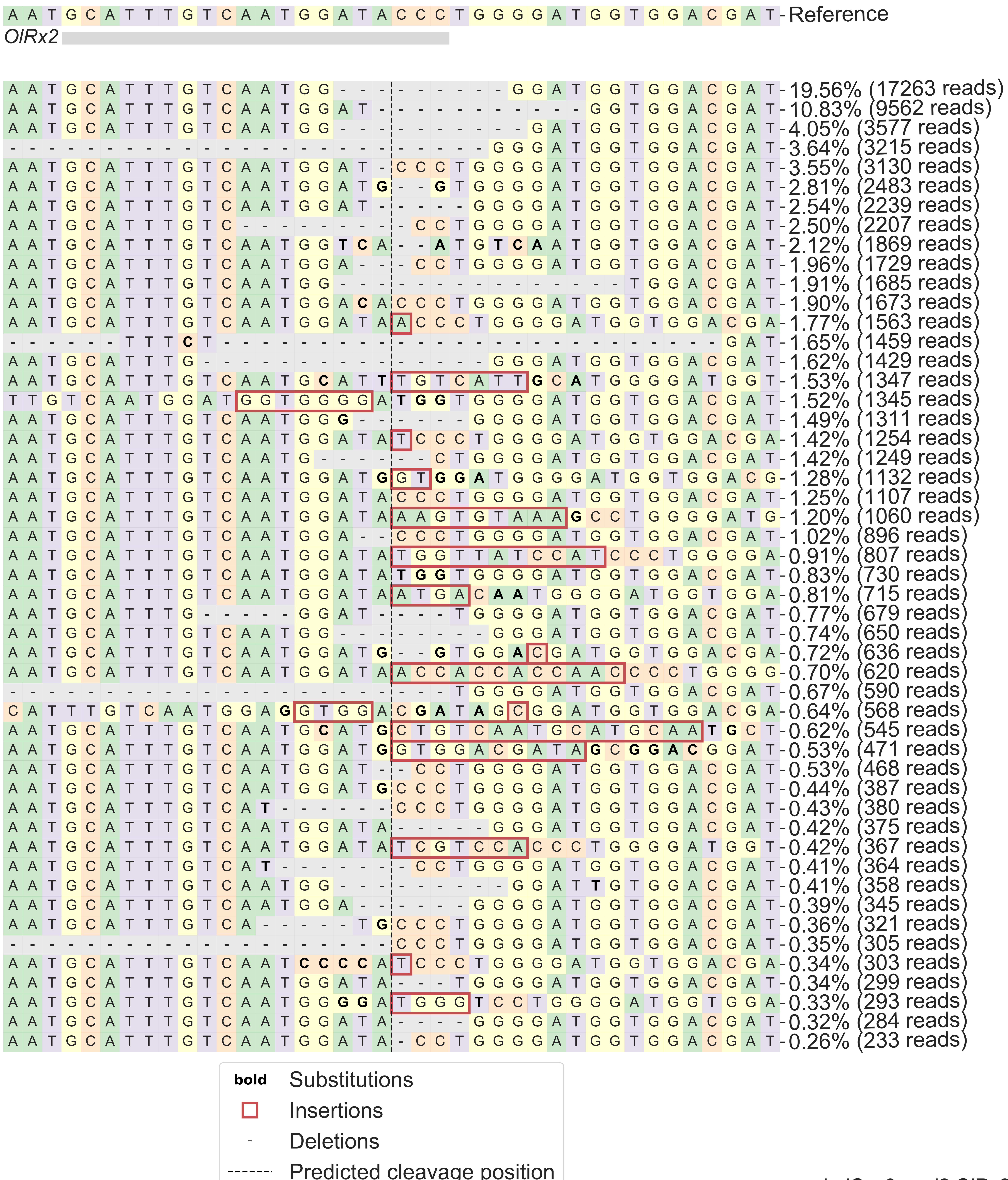

heiCas9 pool2 OlRx2

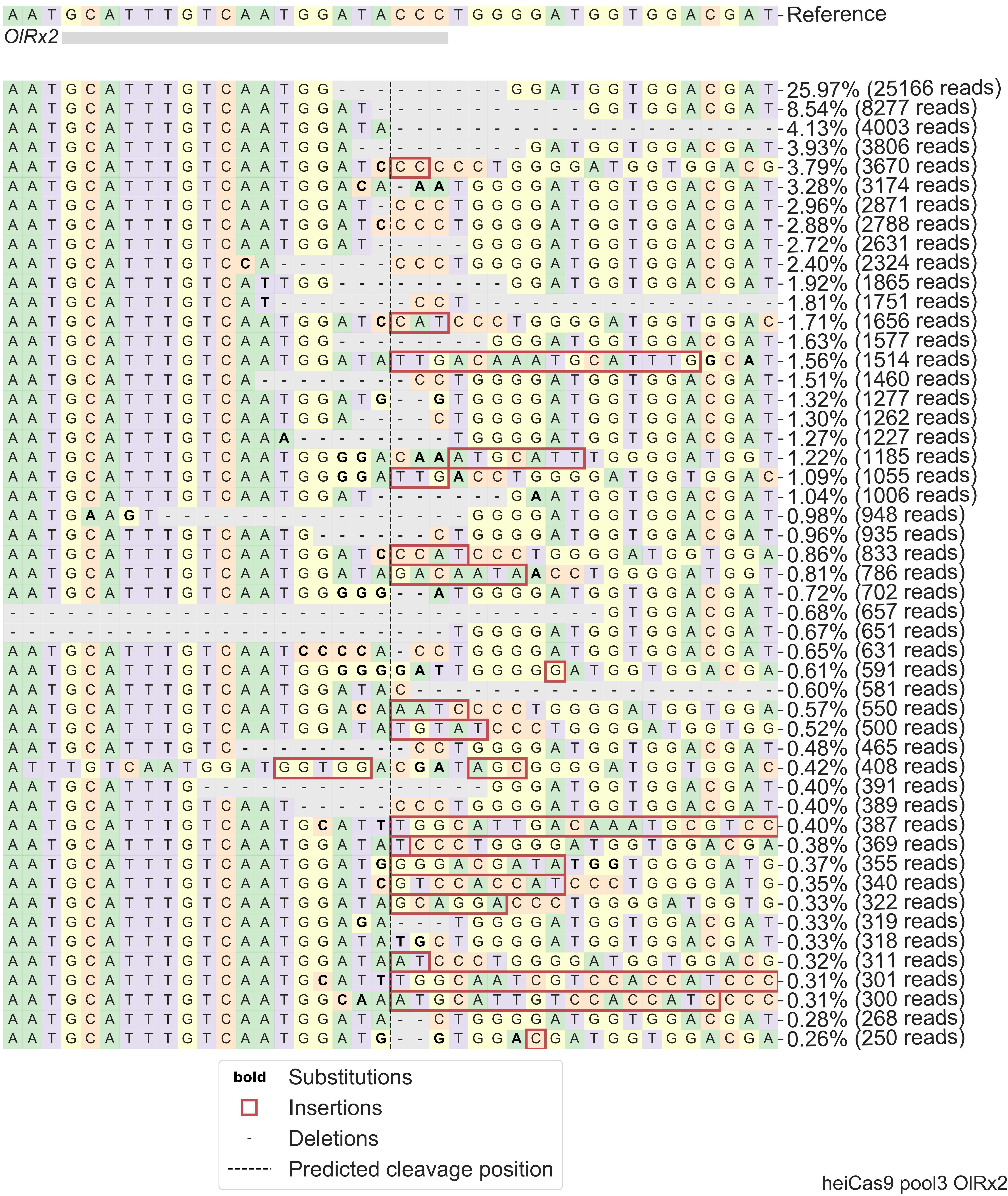

Reference
OlRx2
25.97% (25166 reads)
8.54% (8277 reads)
4.13% (4003 reads)
3.93% (3806 reads)
3.79% (3670 reads)
3.28% (3174 reads)
2.96% (2871 reads)
2.88% (2788 reads)
2.72% (2631 reads)
2.40% (2324 reads)
1.92% (1865 reads)
1.81% (1751 reads)
1.71% (1656 reads)
1.63% (1577 reads)
1.56% (1514 reads)
1.51% (1460 reads)
1.32% (1277 reads)
1.30% (1262 reads)
1.27% (1227 reads)
1.22% (1185 reads)
1.09% (1055 reads)
1.04% (1006 reads)
0.98% (948 reads)
0.96% (935 reads)
0.86% (833 reads)
0.81% (786 reads)
0.72% (702 reads)
0.68% (657 reads)
0.67% (651 reads)
0.65% (631 reads)
0.61% (591 reads)
0.60% (581 reads)
0.57% (550 reads)
0.52% (500 reads)
0.48% (465 reads)
0.42% (408 reads)
0.40% (391 reads)
0.40% (389 reads)
0.40% (387 reads)
0.38% (369 reads)
0.37% (355 reads)
0.35% (340 reads)
0.33% (322 reads)
0.33% (319 reads)
0.33% (318 reads)
0.32% (311 reads)
0.31% (301 reads)
0.31% (300 reads)
0.28% (268 reads)
0.26% (250 reads)
bold Substitutions
Insertions
Deletions
Predicted cleavage position
heiCas9 pool3 OlRx2

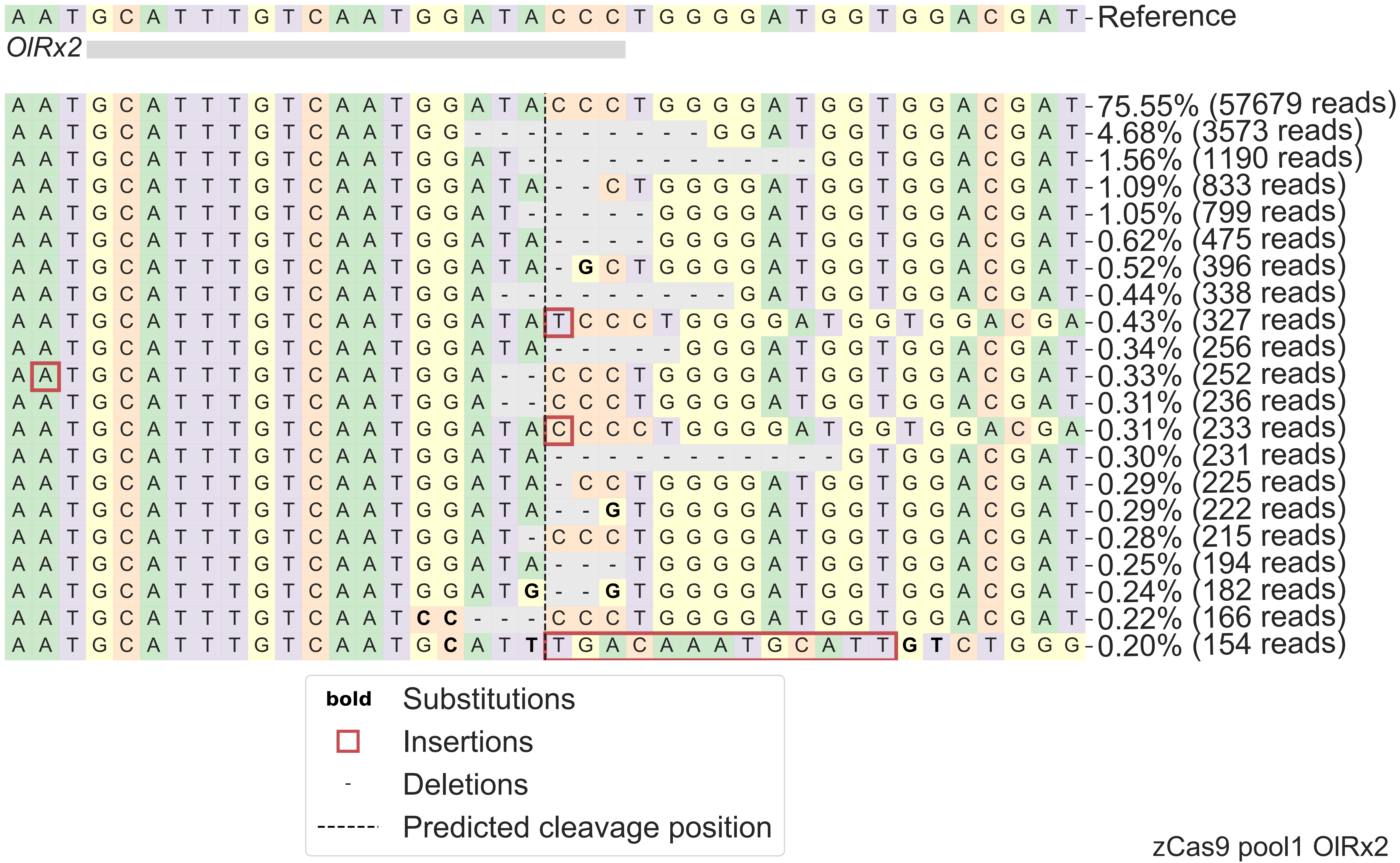
Reference
OlRx2
75.55% (57679 reads)
4.68% (3573 reads)
1.56% (1190 reads)
1.09% (833 reads)
1.05% (799 reads)
0.62% (475 reads)
0.52% (396 reads)
0.44% (338 reads)
0.43% (327 reads)
0.34% (256 reads)
0.33% (252 reads)
0.31% (236 reads)
0.31% (233 reads)
0.30% (231 reads)
0.29% (225 reads)
0.29% (222 reads)
0.28% (215 reads)
0.25% (194 reads)
0.24% (182 reads)
0.22% (166 reads)
0.20% (154 reads)
bold Substitutions
Insertions
Deletions
Predicted cleavage position
zCas9 pool1 OlRx2

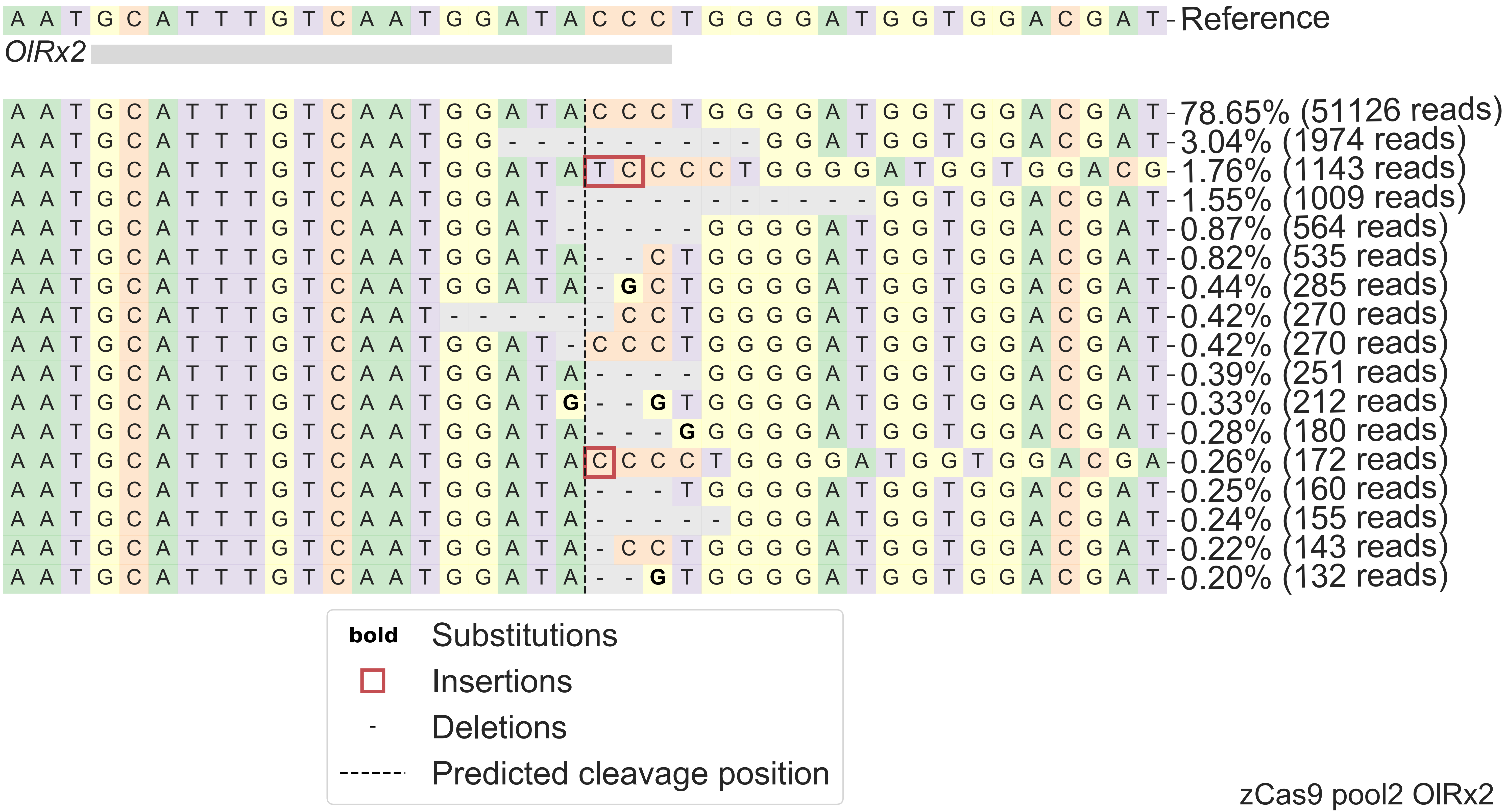

zCas9 pool2 OlRx2

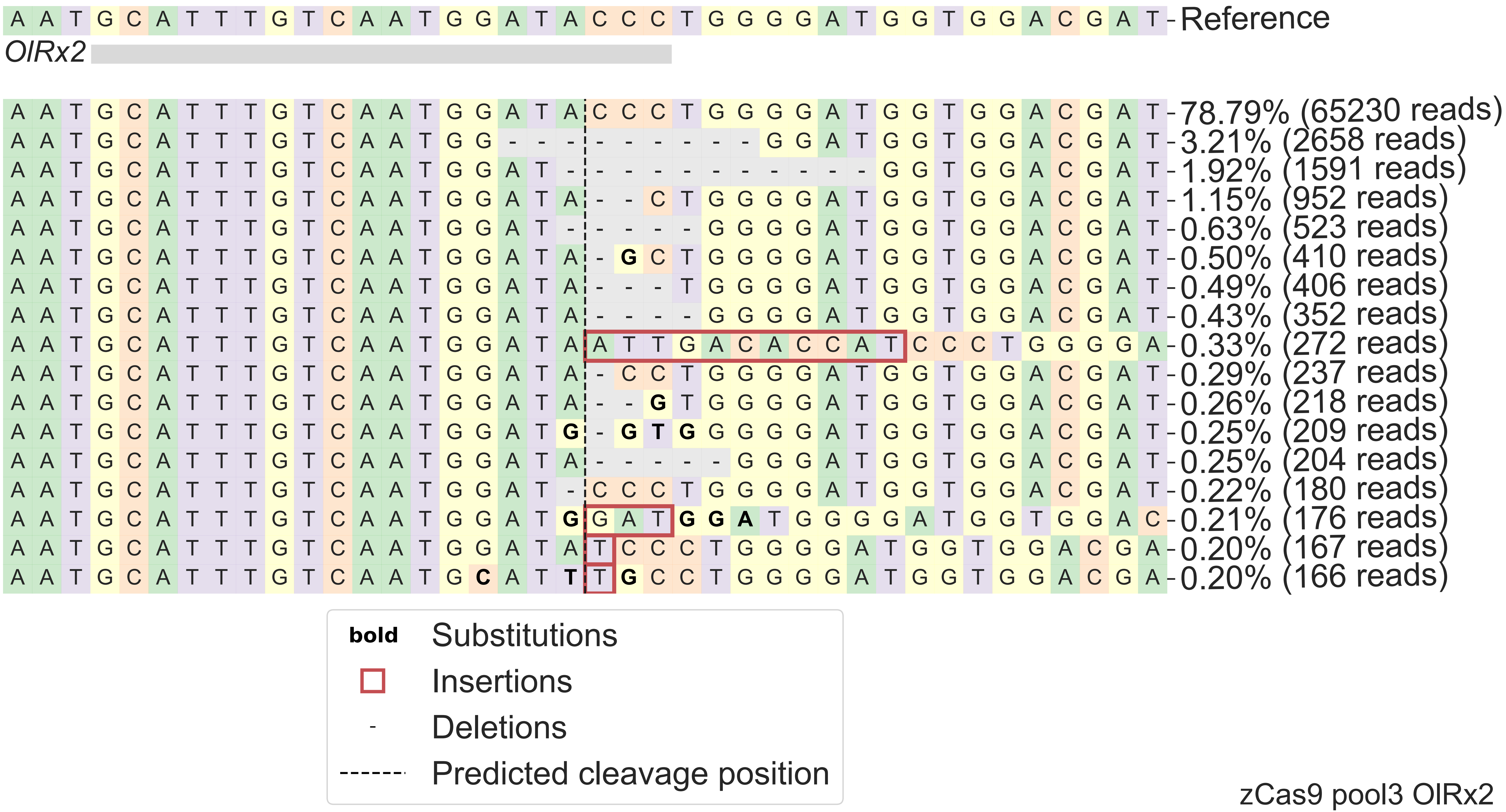
AATGCATTTGTCAATGGATACCCTGGGGATGGTGGACGAT Reference
OlRx2
AATGCATTTGTCAATGGATACCCTGGGGATGGTGGACGAT 78.79% (65230 reads)
AATGCATTTGTCAATGG---------GGATGGTGGACGAT 3.21% (2658 reads)
AATGCATTTGTCAATGGAT-----------GGTGGACGAT 1.92% (1591 reads)
AATGCATTTGTCAATGGATA--CTGGGGATGGTGGACGAT 1.15% (952 reads)
AATGCATTTGTCAATGGAT-----GGGGATGGTGGACGAT 0.63% (523 reads)
AATGCATTTGTCAATGGATA-GCTGGGGATGGTGGACGAT 0.50% (410 reads)
AATGCATTTGTCAATGGATA---TGGGGATGGTGGACGAT 0.49% (406 reads)
AATGCATTTGTCAATGGATA----GGGGATGGTGGACGAT 0.43% (352 reads)
AATGCATTTGTCAATGGATAATTGACACCATCCCTGGGGA 0.33% (272 reads)
AATGCATTTGTCAATGGATA-CCTGGGGATGGTGGACGAT 0.29% (237 reads)
AATGCATTTGTCAATGGATA--GTGGGGATGGTGGACGAT 0.26% (218 reads)
AATGCATTTGTCAATGGATG-GTGGGGGATGGTGGACGAT 0.25% (209 reads)
AATGCATTTGTCAATGGATA-----GGGATGGTGGACGAT 0.25% (204 reads)
AATGCATTTGTCAATGGAT-CCCTGGGGATGGTGGACGAT 0.22% (180 reads)
AATGCATTTGTCAATGGATGGATGGATGGGGATGGTGGAC 0.21% (176 reads)
AATGCATTTGTCAATGGATATCCCTGGGGATGGTGGACGA 0.20% (167 reads)
AATGCATTTGTCAATGCATTTGCCTGGGGATGGTGGACGA 0.20% (166 reads)
bold Substitutions
Insertions
- Deletions
Predicted cleavage position
zCas9 pool3 OlRx2

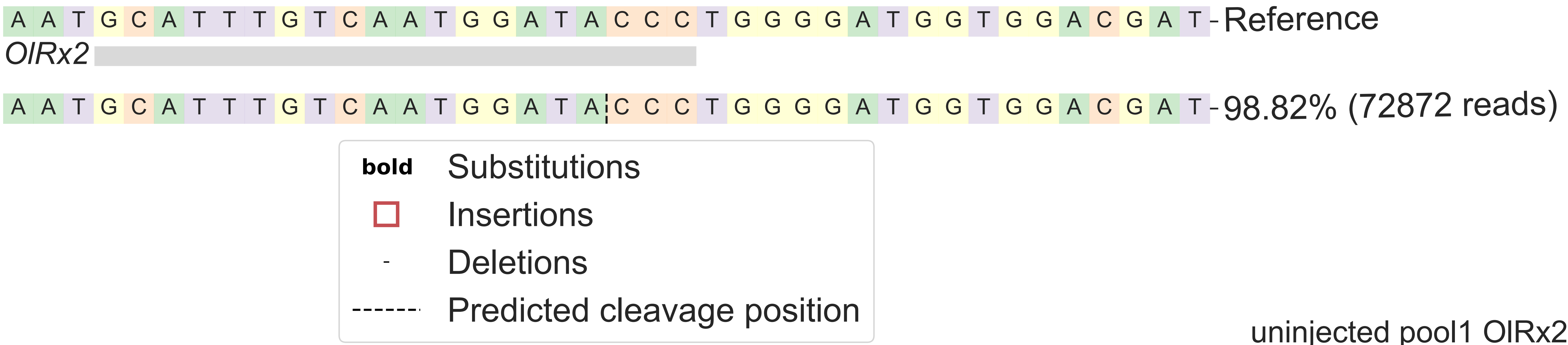
A A T G C A T T T G T C A A T G G A T A C C C T G G G G A T G G T G G A C G A T - Reference
OlRx2
A A T G C A T T T G T C A A T G G A T A C C C T G G G G A T G G T G G A C G A T - 98.82% (72872 reads)
bold Substitutions
Insertions
Deletions
Predicted cleavage position
uninjected pool1 OlRx2

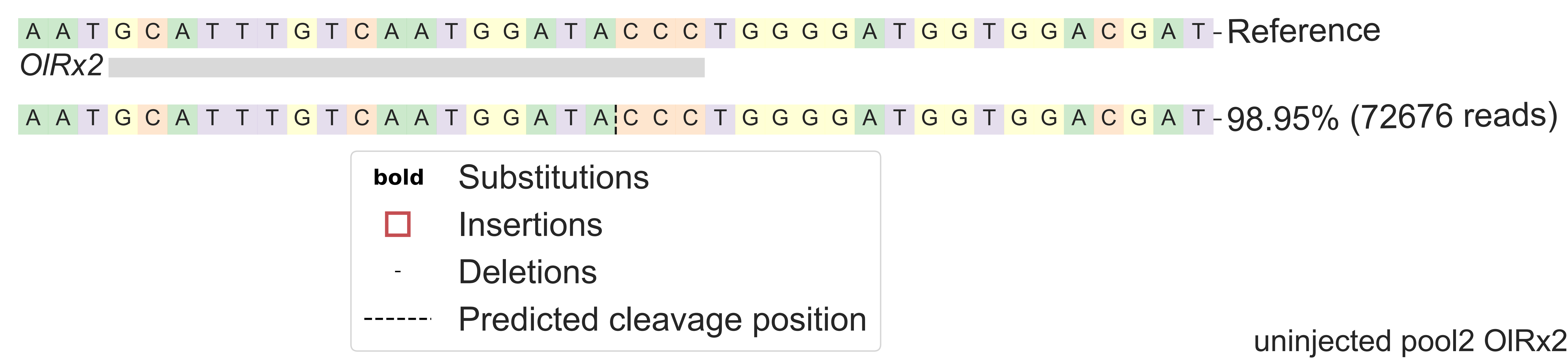
A A T G C A T T T G T C A A T G G A T A C C C T G G G G A T G G T G G A C G A T - Reference
OlRx2
A A T G C A T T T G T C A A T G G A T A C C C T G G G G A T G G T G G A C G A T - 98.95% (72676 reads)
bold Substitutions
Insertions
- Deletions
Predicted cleavage position
uninjected pool2 OlRx2

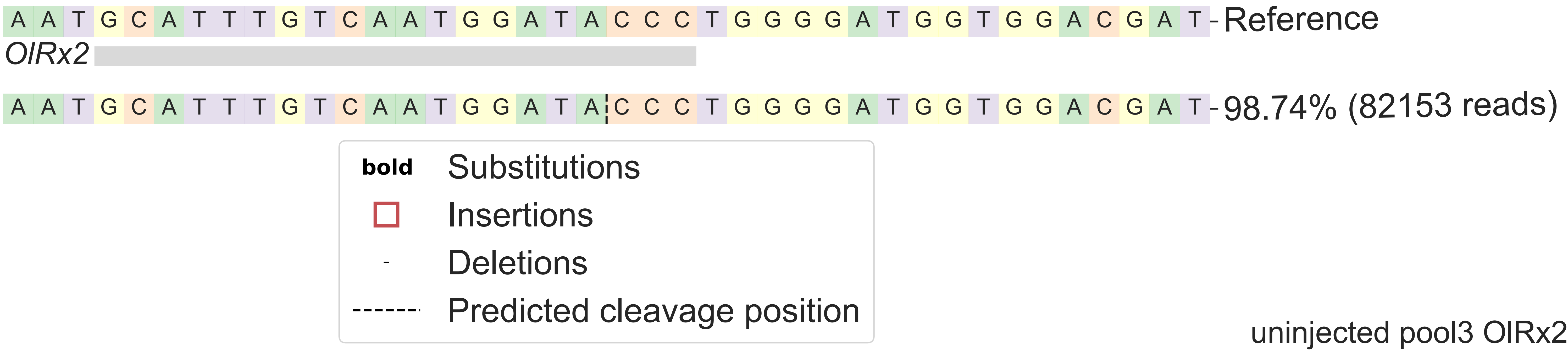
A A T G C A T T T G T C A A T G G A T A C C C T G G G G A T G G T G G A C G A T - Reference
OlRx2
A A T G C A T T T G T C A A T G G A T A C C C T G G G G A T G G T G G A C G A T - 98.74% (82153 reads)
bold Substitutions
Insertions
- Deletions
Predicted cleavage position
uninjected pool3 OlRx2

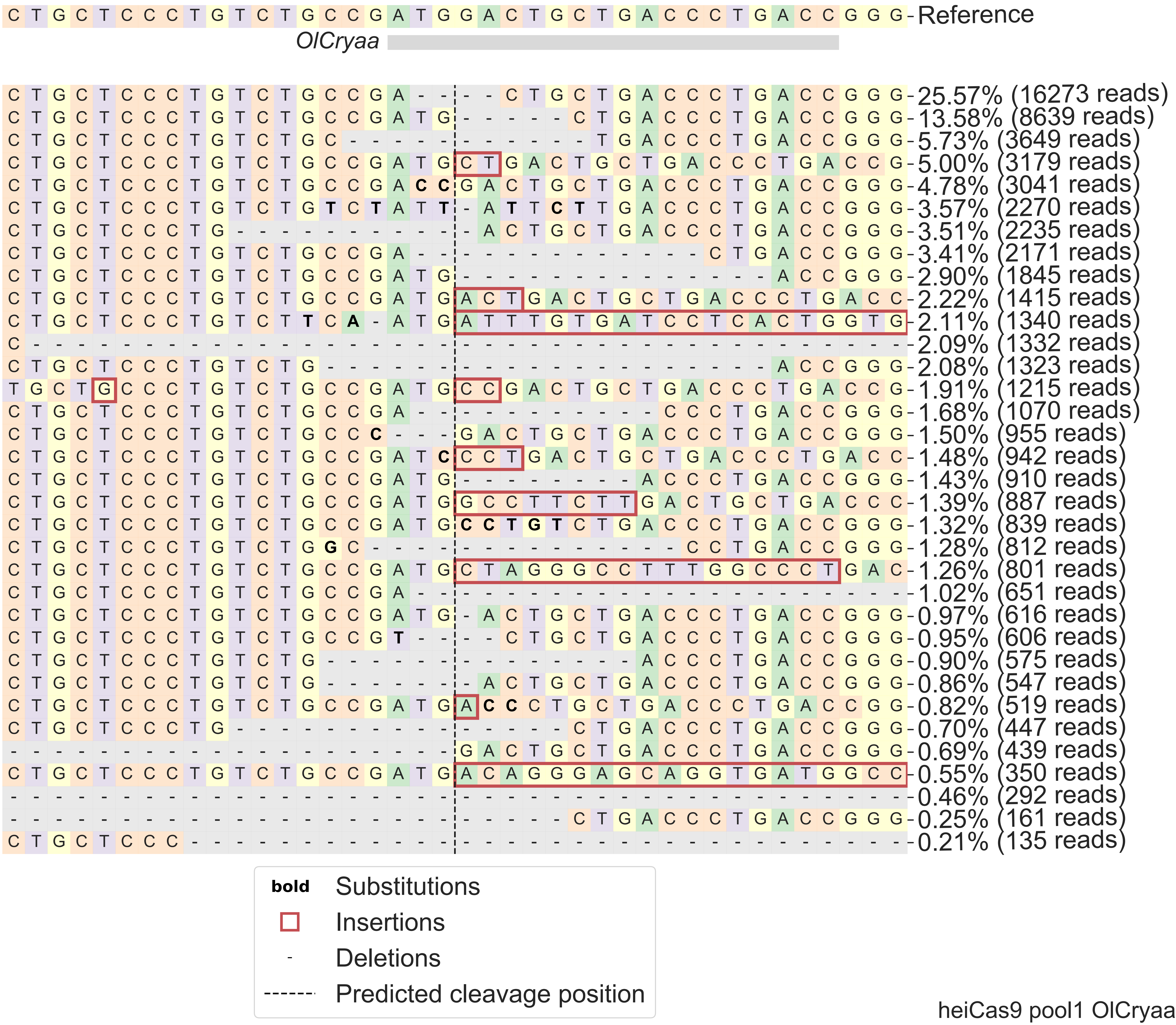
C T G C T C C C T G T C T G C C G A T G G A C T G C T G A C C C T G A C C G G G
Reference
OlCryaa
25.57% (16273 reads)
13.58% (8639 reads)
5.73% (3649 reads)
5.00% (3179 reads)
4.78% (3041 reads)
3.57% (2270 reads)
3.51% (2235 reads)
3.41% (2171 reads)
2.90% (1845 reads)
2.22% (1415 reads)
2.11% (1340 reads)
2.09% (1332 reads)
2.08% (1323 reads)
1.91% (1215 reads)
1.68% (1070 reads)
1.50% (955 reads)
1.48% (942 reads)
1.43% (910 reads)
1.39% (887 reads)
1.32% (839 reads)
1.28% (812 reads)
1.26% (801 reads)
1.02% (651 reads)
0.97% (616 reads)
0.95% (606 reads)
0.90% (575 reads)
0.86% (547 reads)
0.82% (519 reads)
0.70% (447 reads)
0.69% (439 reads)
0.55% (350 reads)
0.46% (292 reads)
0.25% (161 reads)
0.21% (135 reads)
bold Substitutions
Insertions
Deletions
Predicted cleavage position
heiCas9 pool1 OlCryaa

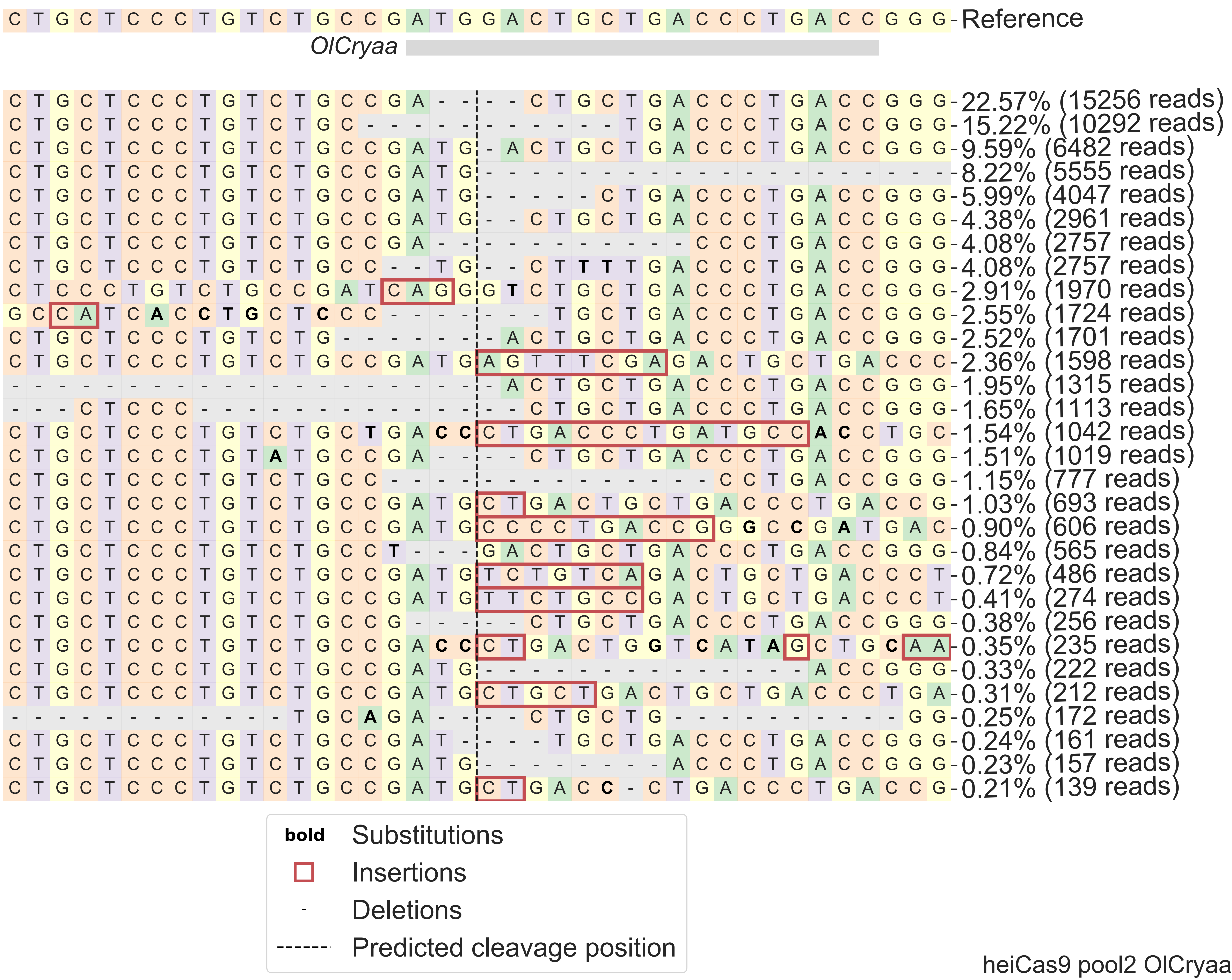

Reference
OlCryaa
22.57% (15256 reads)
15.22% (10292 reads)
9.59% (6482 reads)
8.22% (5555 reads)
5.99% (4047 reads)
4.38% (2961 reads)
4.08% (2757 reads)
4.08% (2757 reads)
2.91% (1970 reads)
2.55% (1724 reads)
2.52% (1701 reads)
2.36% (1598 reads)
1.95% (1315 reads)
1.65% (1113 reads)
1.54% (1042 reads)
1.51% (1019 reads)
1.15% (777 reads)
1.03% (693 reads)
0.90% (606 reads)
0.84% (565 reads)
0.72% (486 reads)
0.41% (274 reads)
0.38% (256 reads)
0.35% (235 reads)
0.33% (222 reads)
0.31% (212 reads)
0.25% (172 reads)
0.24% (161 reads)
0.23% (157 reads)
0.21% (139 reads)
bold Substitutions
Insertions
Deletions
Predicted cleavage position
heiCas9 pool2 OlCryaa

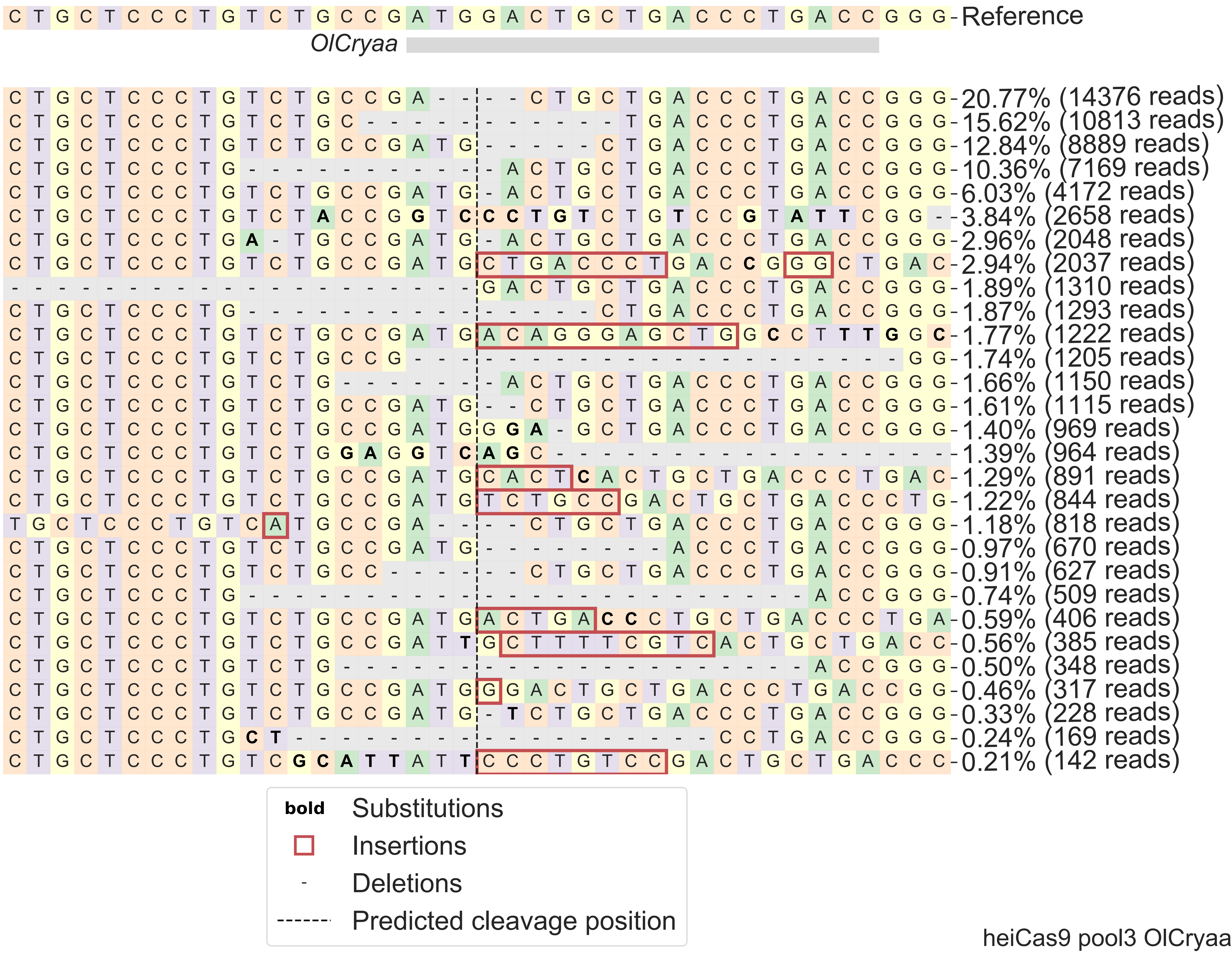
CTGCTCCCTGTCTGCCGATGGACTGCTGACCCTGACCGGG Reference
OlCryaa
20.77% (14376 reads)
15.62% (10813 reads)
12.84% (8889 reads)
10.36% (7169 reads)
6.03% (4172 reads)
3.84% (2658 reads)
2.96% (2048 reads)
2.94% (2037 reads)
1.89% (1310 reads)
1.87% (1293 reads)
1.77% (1222 reads)
1.74% (1205 reads)
1.66% (1150 reads)
1.61% (1115 reads)
1.40% (969 reads)
1.39% (964 reads)
1.29% (891 reads)
1.22% (844 reads)
1.18% (818 reads)
0.97% (670 reads)
0.91% (627 reads)
0.74% (509 reads)
0.59% (406 reads)
0.56% (385 reads)
0.50% (348 reads)
0.46% (317 reads)
0.33% (228 reads)
0.24% (169 reads)
0.21% (142 reads)
bold Substitutions
Insertions
Deletions
Predicted cleavage position
heiCas9 pool3 OlCryaa

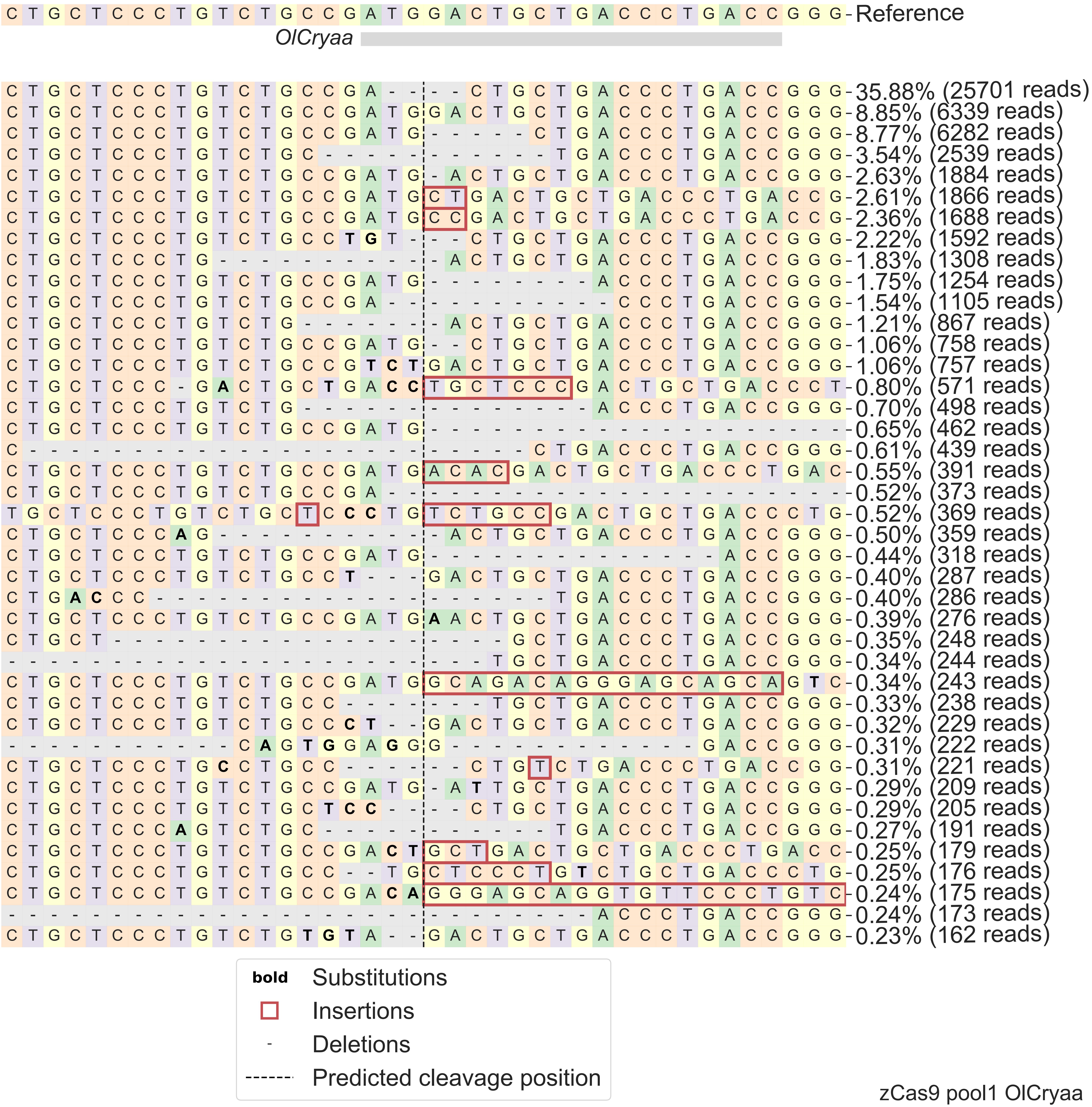

zCas9 pool1 OlCryaa

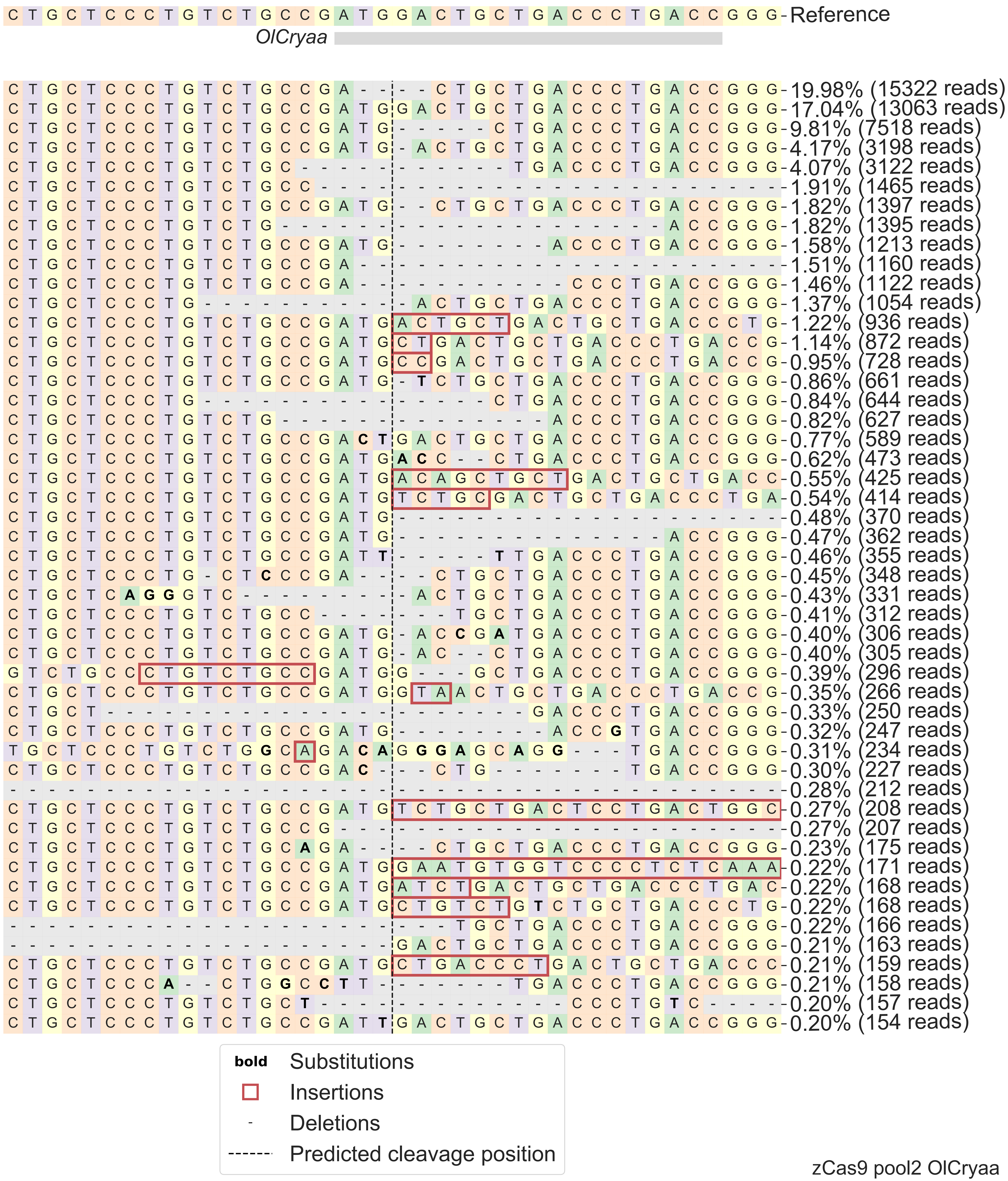
C T G C T C C C T G T C T G C C G A T G G A C T G C T G A C C C T G A C C G G G Reference
OlCryaa
19.98% (15322 reads)
17.04% (13063 reads)
9.81% (7518 reads)
4.17% (3198 reads)
4.07% (3122 reads)
1.91% (1465 reads)
1.82% (1397 reads)
1.82% (1395 reads)
1.58% (1213 reads)
1.51% (1160 reads)
1.46% (1122 reads)
1.37% (1054 reads)
1.22% (936 reads)
1.14% (872 reads)
0.95% (728 reads)
0.86% (661 reads)
0.84% (644 reads)
0.82% (627 reads)
0.77% (589 reads)
0.62% (473 reads)
0.55% (425 reads)
0.54% (414 reads)
0.48% (370 reads)
0.47% (362 reads)
0.46% (355 reads)
0.45% (348 reads)
0.43% (331 reads)
0.41% (312 reads)
0.40% (306 reads)
0.40% (305 reads)
0.39% (296 reads)
0.35% (266 reads)
0.33% (250 reads)
0.32% (247 reads)
0.31% (234 reads)
0.30% (227 reads)
0.28% (212 reads)
0.27% (208 reads)
0.27% (207 reads)
0.23% (175 reads)
0.22% (171 reads)
0.22% (168 reads)
0.22% (168 reads)
0.22% (166 reads)
0.21% (163 reads)
0.21% (159 reads)
0.21% (158 reads)
0.20% (157 reads)
0.20% (154 reads)
bold Substitutions
Insertions
Deletions
Predicted cleavage position
zCas9 pool2 OlCryaa

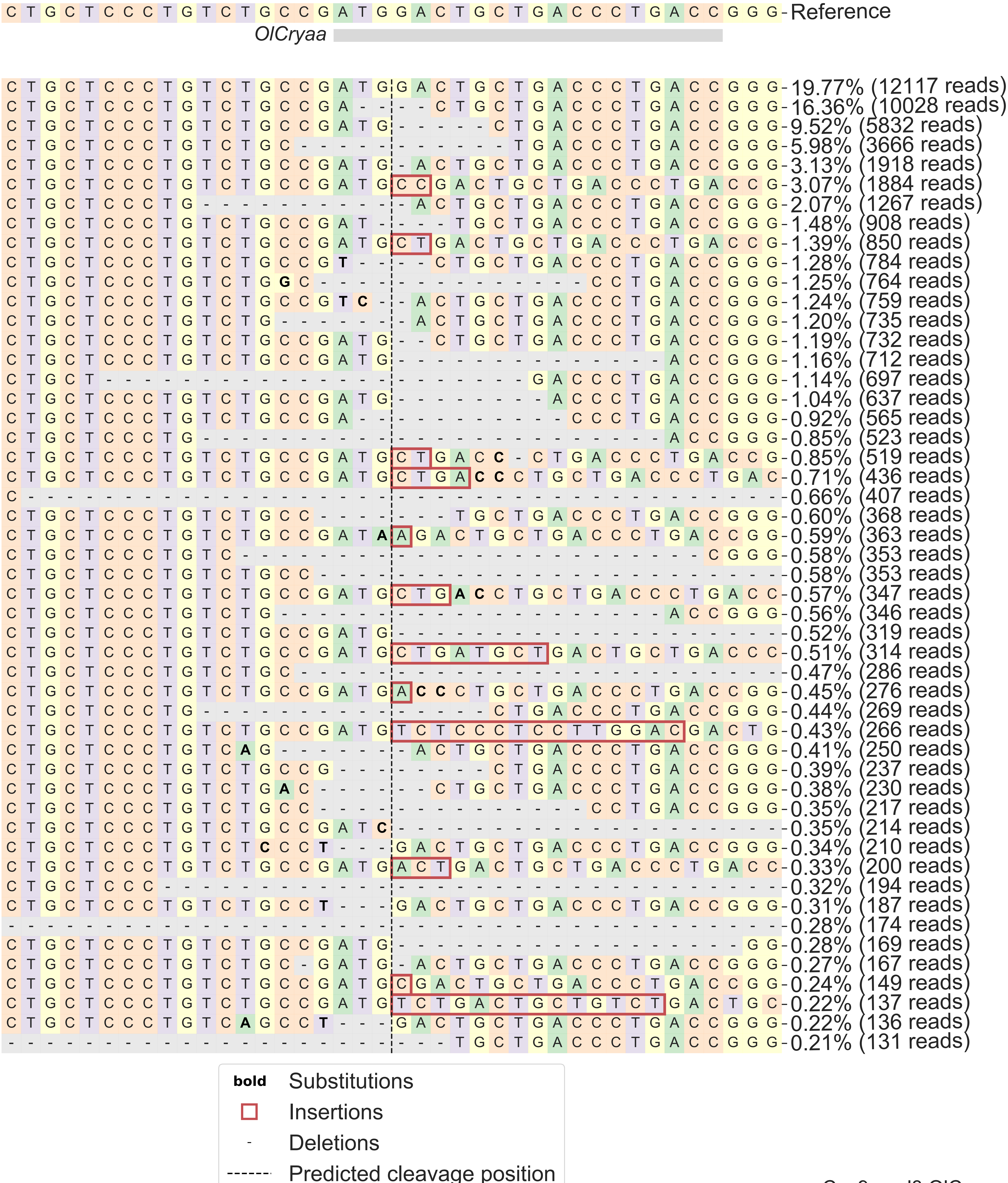

zCas9 pool3 OlCryaa

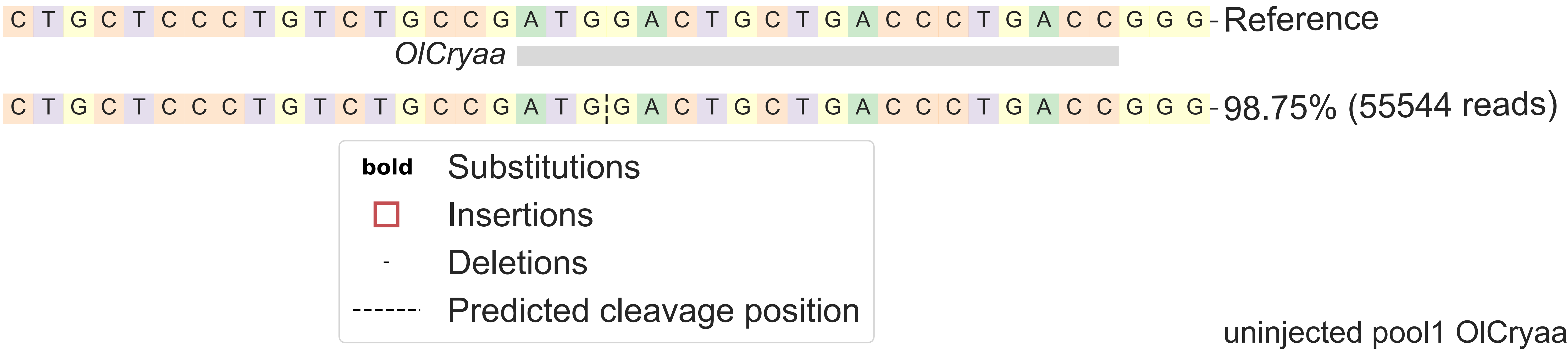

C T G C T C C C T G T C T G C C G A T G G A C T G C T G A C C C T G A C C G G G
Reference
OlCryaa
C T G C T C C C T G T C T G C C G A T G G A C T G C T G A C C C T G A C C G G G
98.75% (55544 reads)
bold Substitutions
Insertions
Deletions
Predicted cleavage position
uninjected pool1 OlCryaa

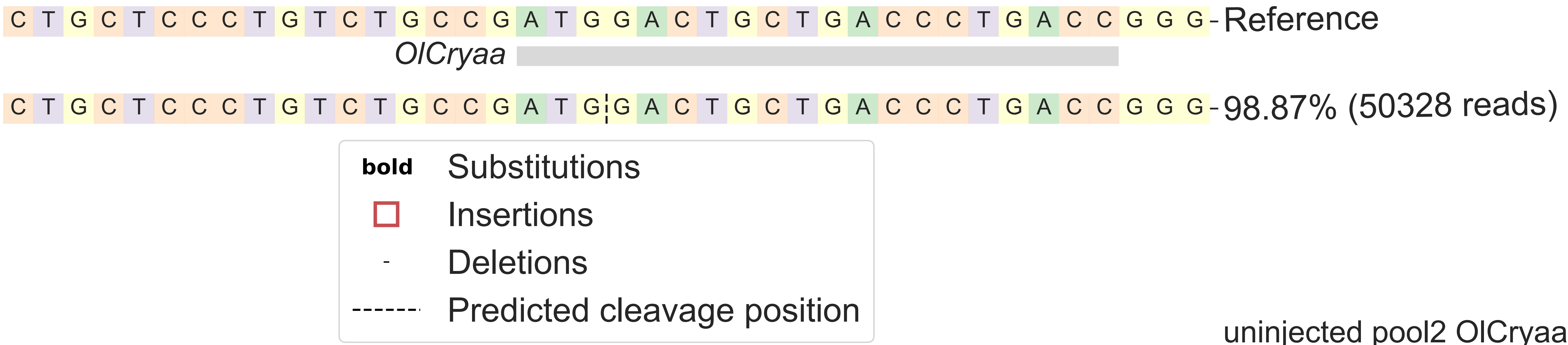

C T G C T C C C T G T C T G C C G A T G G A C T G C T G A C C C T G A C C G G G - Reference
OlCryaa
C T G C T C C C T G T C T G C C G A T G G A C T G C T G A C C C T G A C C G G G - 98.87% (50328 reads)
bold Substitutions
Insertions
- Deletions
Predicted cleavage position
uninjected pool2 OlCryaa

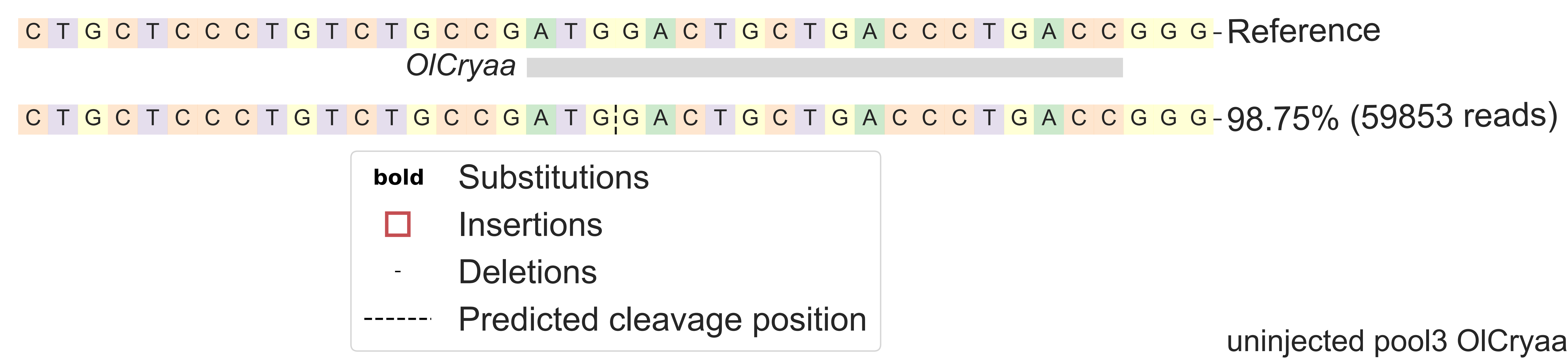

CTGCTCCCTGTCTGCCGATGGACTGCTGACCCTGACCGGG-
Reference
OlCryaa
CTGCTCCCTGTCTGCCGATGGACTGCTGACCCTGACCGGG-
98.75% (59853 reads)
bold Substitutions
Insertions
Deletions
Predicted cleavage position
uninjected pool3 OlCryaa

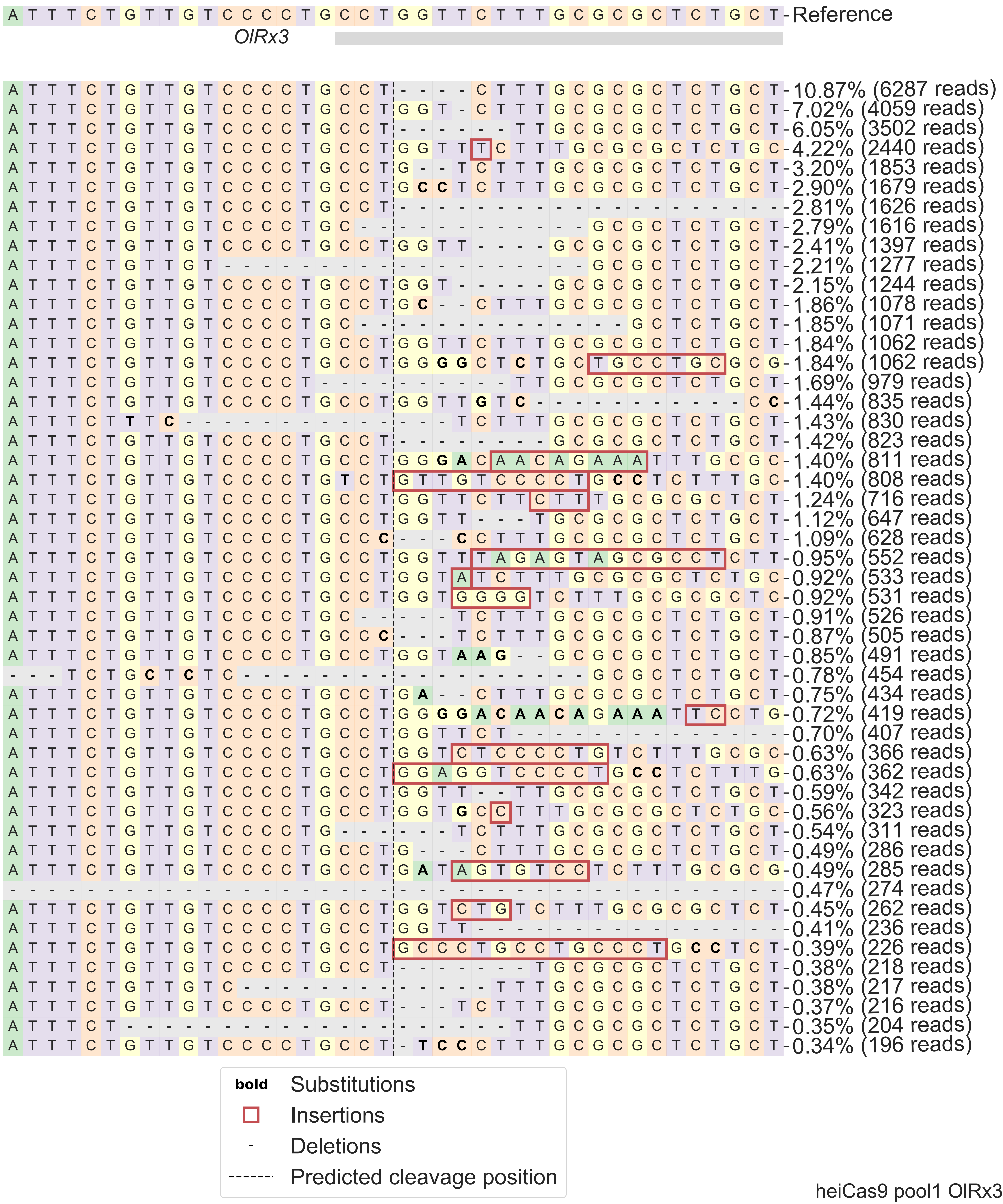

heiCas9 pool1 OlRx3

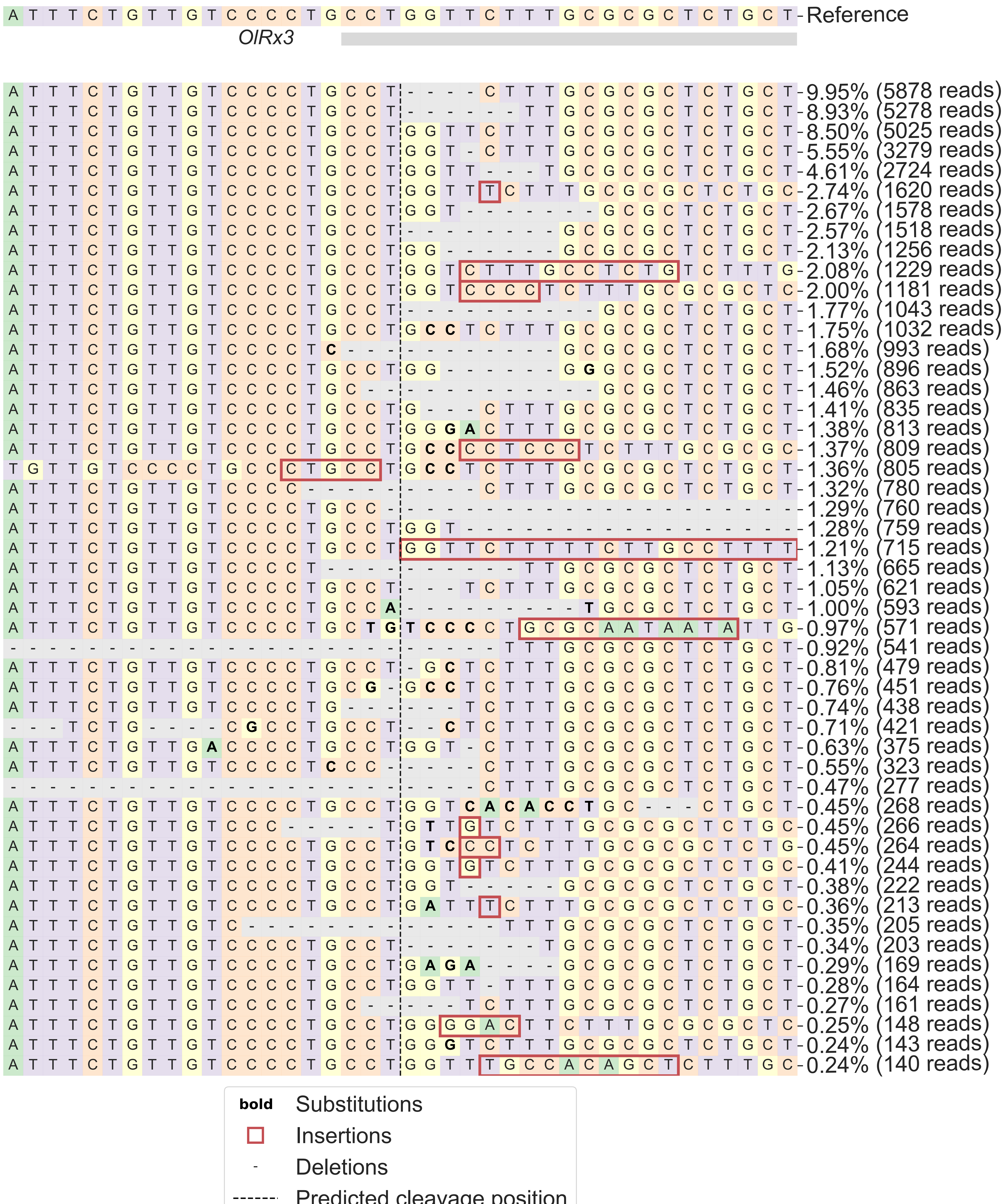

heiCas9 pool2 OlRx3

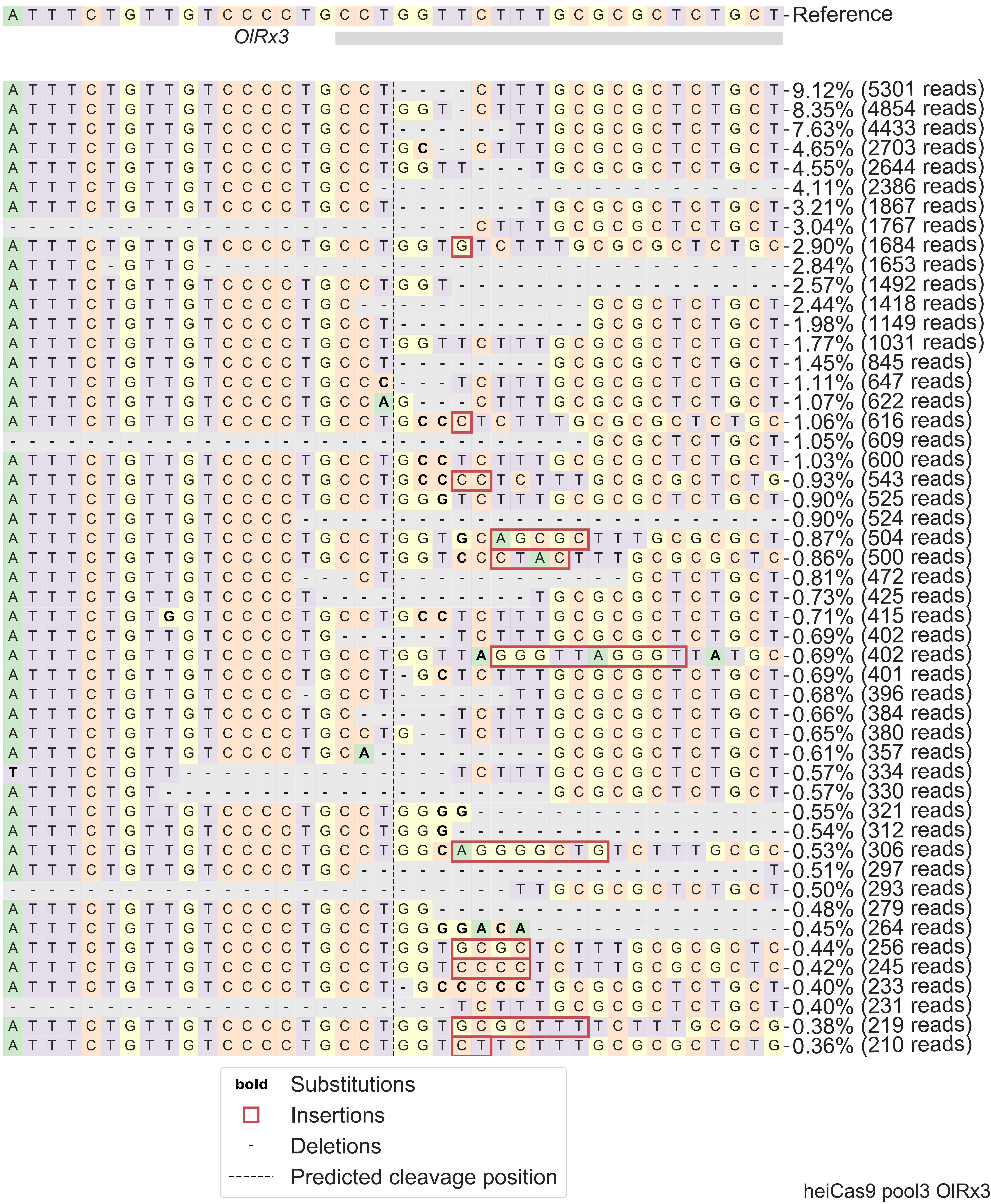

heiCas9 pool3 OlRx3

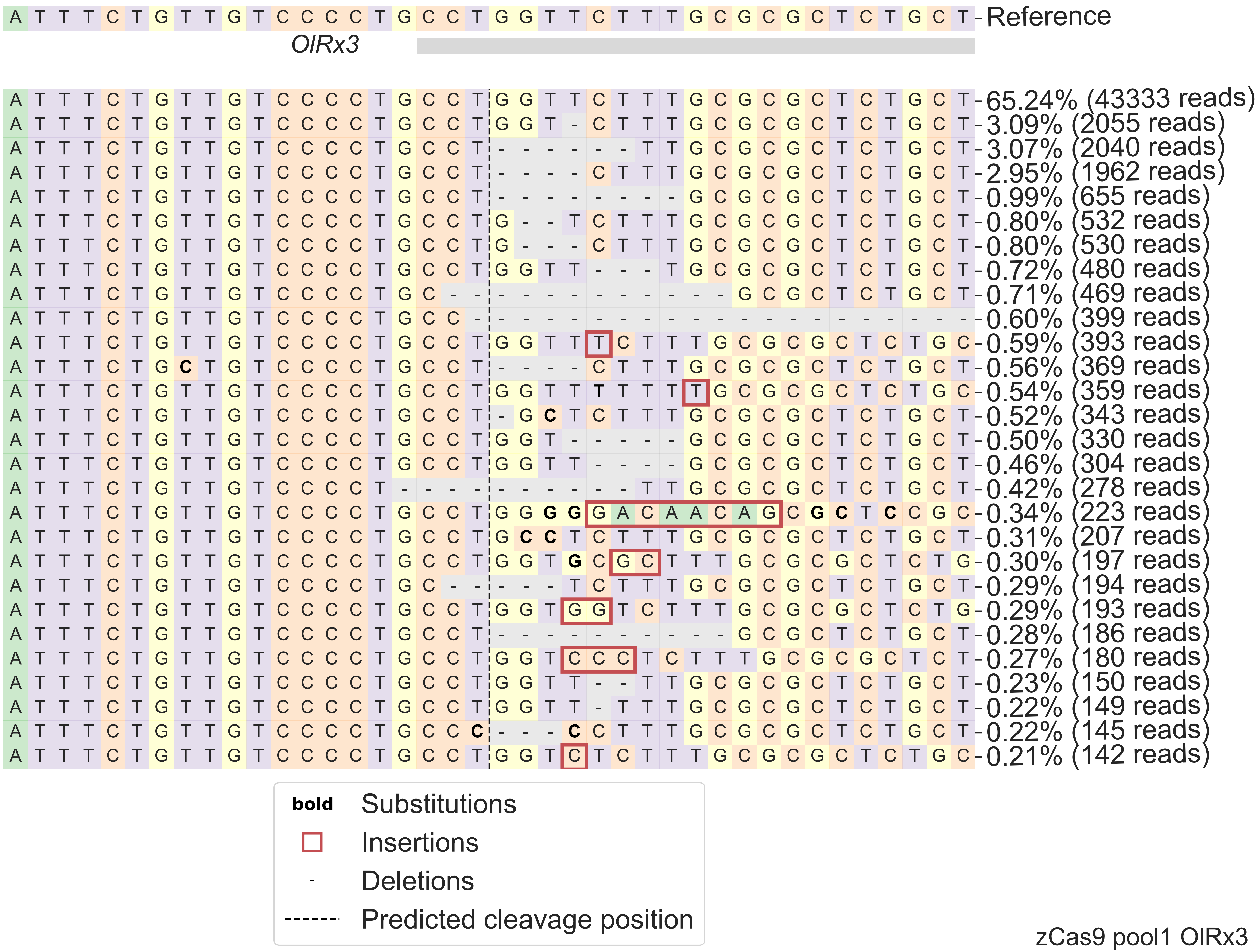
Reference
OlRx3
65.24% (43333 reads)
3.09% (2055 reads)
3.07% (2040 reads)
2.95% (1962 reads)
0.99% (655 reads)
0.80% (532 reads)
0.80% (530 reads)
0.72% (480 reads)
0.71% (469 reads)
0.60% (399 reads)
0.59% (393 reads)
0.56% (369 reads)
0.54% (359 reads)
0.52% (343 reads)
0.50% (330 reads)
0.46% (304 reads)
0.42% (278 reads)
0.34% (223 reads)
0.31% (207 reads)
0.30% (197 reads)
0.29% (194 reads)
0.29% (193 reads)
0.28% (186 reads)
0.27% (180 reads)
0.23% (150 reads)
0.22% (149 reads)
0.22% (145 reads)
0.21% (142 reads)
bold Substitutions
Insertions
- Deletions
Predicted cleavage position
zCas9 pool1 OlRx3

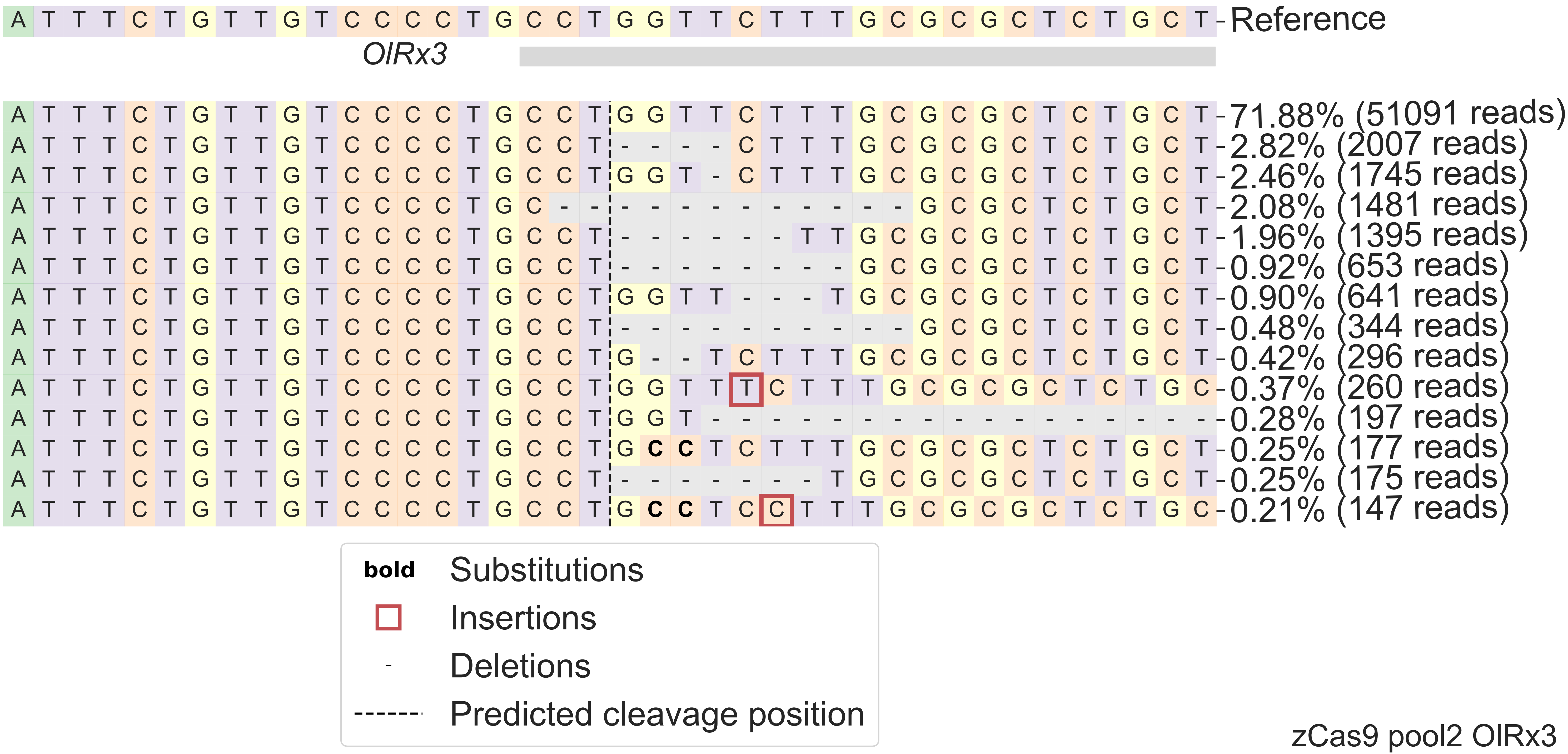
Reference
OlRx3
71.88% (51091 reads)
2.82% (2007 reads)
2.46% (1745 reads)
2.08% (1481 reads)
1.96% (1395 reads)
0.92% (653 reads)
0.90% (641 reads)
0.48% (344 reads)
0.42% (296 reads)
0.37% (260 reads)
0.28% (197 reads)
0.25% (177 reads)
0.25% (175 reads)
0.21% (147 reads)
bold Substitutions
Insertions
Deletions
Predicted cleavage position
zCas9 pool2 OlRx3

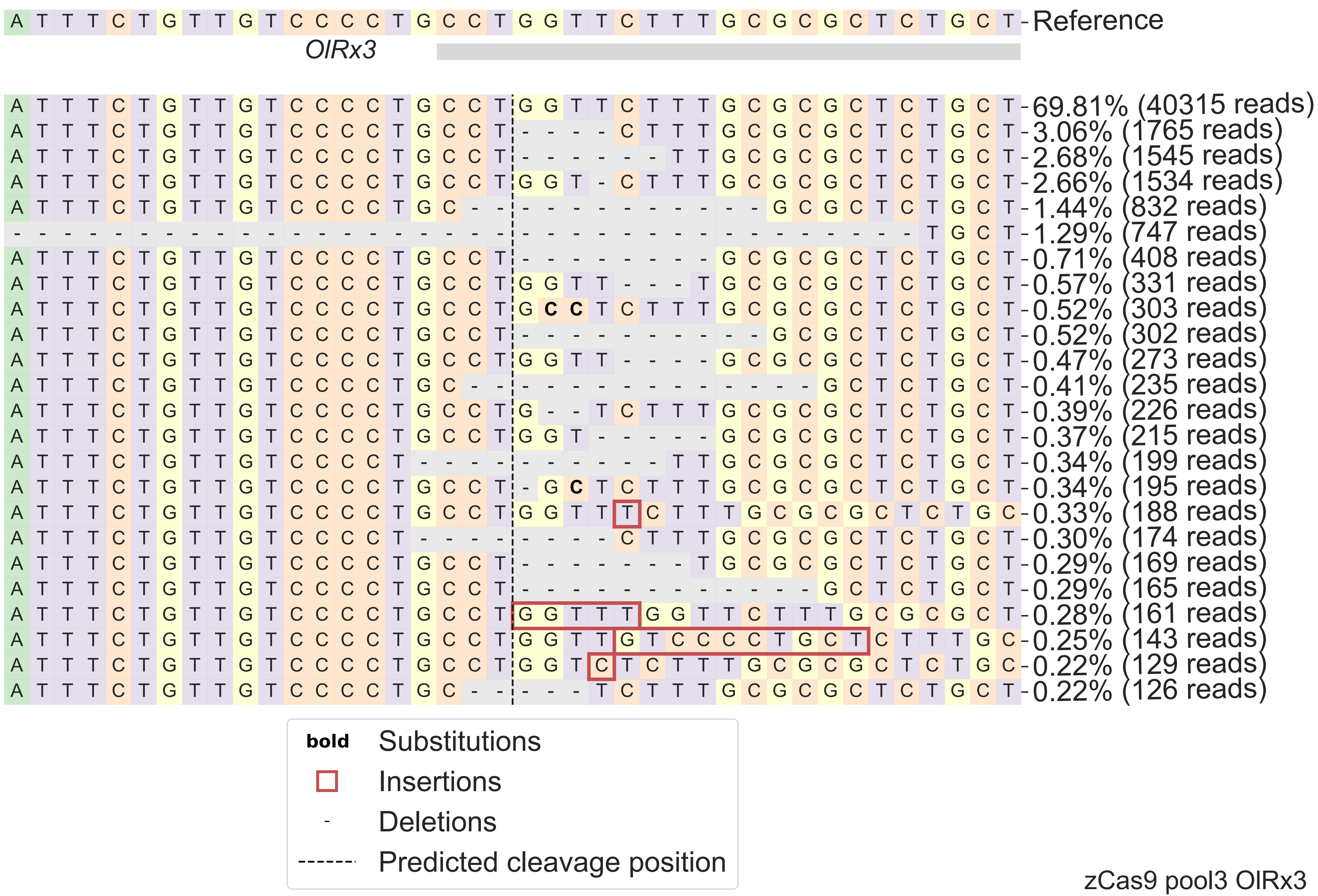

Reference
OlRx3
69.81% (40315 reads)
3.06% (1765 reads)
2.68% (1545 reads)
2.66% (1534 reads)
1.44% (832 reads)
1.29% (747 reads)
0.71% (408 reads)
0.57% (331 reads)
0.52% (303 reads)
0.52% (302 reads)
0.47% (273 reads)
0.41% (235 reads)
0.39% (226 reads)
0.37% (215 reads)
0.34% (199 reads)
0.34% (195 reads)
0.33% (188 reads)
0.30% (174 reads)
0.29% (169 reads)
0.29% (165 reads)
0.28% (161 reads)
0.25% (143 reads)
0.22% (129 reads)
0.22% (126 reads)
bold Substitutions
Insertions
- Deletions
Predicted cleavage position
zCas9 pool3 OlRx3

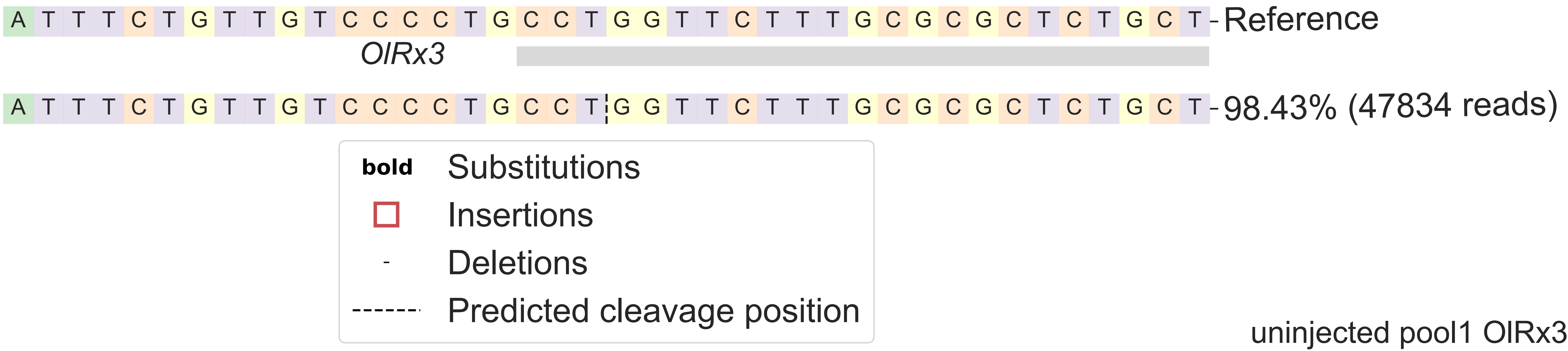
A T T T C T G T T G T C C C C T G C C T G G T T C T T T G C G C G C T C T G C T - Reference
OlRx3
A T T T C T G T T G T C C C C T G C C T G G T T C T T T G C G C G C T C T G C T - 98.43% (47834 reads)
bold Substitutions
Insertions
Deletions
Predicted cleavage position
uninjected pool1 OlRx3

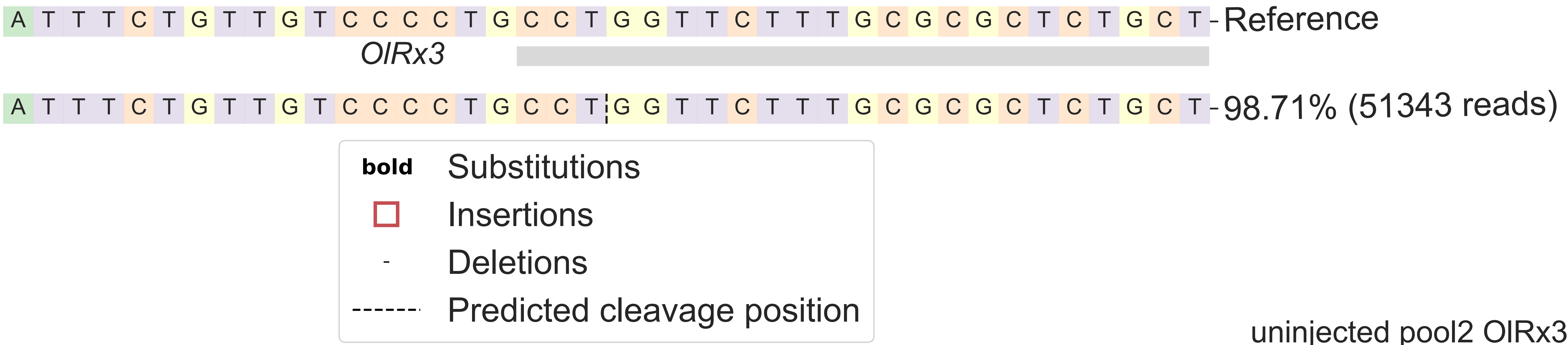
A T T T C T G T T G T C C C C T G C C T G G T T C T T T G C G C G C T C T G C T - Reference
OlRx3
A T T T C T G T T G T C C C C T G C C T G G T T C T T T G C G C G C T C T G C T - 98.71% (51343 reads)
bold Substitutions
Insertions
- Deletions
Predicted cleavage position
uninjected pool2 OlRx3

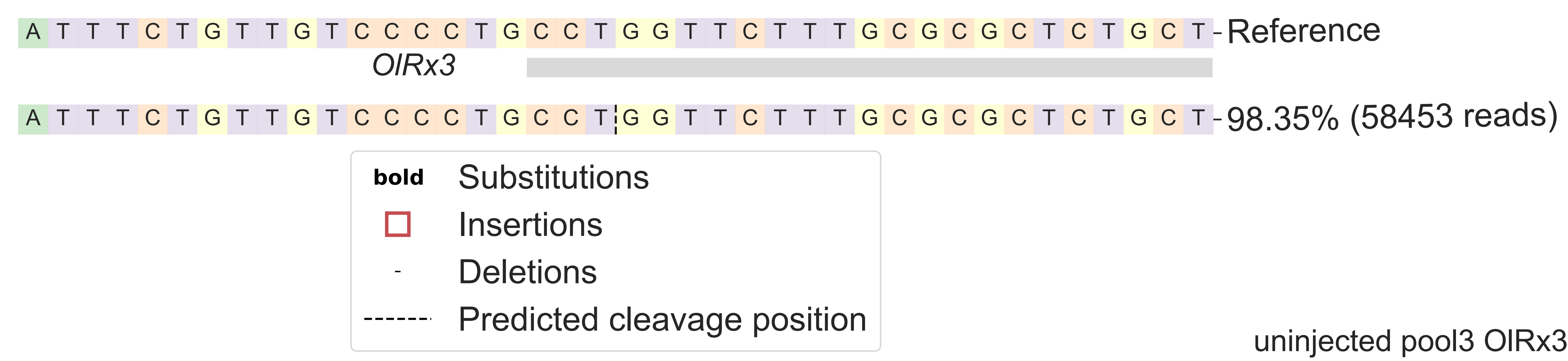
A T T T C T G T T G T C C C C T G C C T G G T T C T T T G C G C G C T C T G C T - Reference
OlRx3
A T T T C T G T T G T C C C C T G C C T G G T T C T T T G C G C G C T C T G C T - 98.35% (58453 reads)
bold Substitutions
Insertions
- Deletions
Predicted cleavage position
uninjected pool3 OlRx3
